# Curcumin mitigated aflatoxin B_1_-induced endoplasmic reticulum stress and gut-kidney axis damage in sheep by regulating the ATF6/GRP78 and IL-1β/NF-κB signaling pathways

**DOI:** 10.1186/s40104-026-01382-2

**Published:** 2026-04-21

**Authors:** Ting Wang, Chuanqi Wang, Tongwei Liu, Jiefeng Li, Hengtong Fang, Jing Zhang

**Affiliations:** 1 College of Animal Sciences, Jilin University, Changchun, 130062 China; 2 National Grain Industry Technology Innovation Center (Medicinal Functional Resources Development), Academy of National Food and Strategic Reserves Administration, Beijing, 100037 China

**Keywords:** Aflatoxin B_1_, Antioxidants, Curcumin, Endoplasmic reticulum stress, H_2_O_2_, Intestine microbiota, Intestine-kidney axis, Sheep

## Abstract

**Background:**

Aflatoxin B_1_ (AFB_1_), a toxic secondary metabolite produced by *Aspergillus flavus* and *Aspergillus parasiticus*, is widely present in various crops and induces endoplasmic reticulum stress in the intestine and kidney of animals, leading to apoptosis and inflammatory damage. Curcumin is a natural phenolic antioxidant that has antioxidant, anti-apoptotic and anti-inflammatory effects. However, the role and mechanism of curcumin in alleviating the toxicity of AFB_1_ in sheep remain unclear. Therefore, this study aimed to investigate the mitigating effects of curcumin on intestinal microbiota disorders and intestinal and kidney injuries in AFB_1_-exposed sheep. Eighteen sheep were randomly divided into three treatment groups. The groups were the control group (CON, basal diet), the AFB_1_ group (AFB_1_, basic diet + 500 μg/kg DM AFB_1_), and the AFB_1__Curcumin group (AFB_1__CUR, basic diet + 500 μg/kg DM AFB_1_ + 800 mg/kg DM curcumin) for 21 d.

**Results:**

AFB_1_ induced intestinal barrier dysfunction, intestinal flora imbalance, and intestinal mucosal damage. Curcumin addition inhibited the activity of the ATF6/GRP78 and IL-1β/NF-κB signaling pathways to alleviate kidney injury and activated the NRF2/KEAP1 pathway and antioxidant system to reduce the toxic substances cycle in the intestine-kidney axis (*P* < 0.05). The protective effects of curcumin on the intestine and kidney are related to a reduction in the levels of *Prevotella* *ruminicola* and *Ruminococcus* *albus*. Therefore, the structure of the microbiota and antioxidant functions were improved, mitigating damage to the intestine-kidney axis.

**Conclusions:**

Curcumin can alleviate AFB_1_-induced disorder of the intestinal microbiota by enhancing intestinal barrier function; reducing intestinal apoptosis, oxidative stress, and inflammatory damage; and regulating the intestinal microbiota via the intestine-kidney axis. Moreover, the activity of the ATF6/GRP78 and IL-1β/NF-κB signaling pathways was inhibited by curcumin to mitigate intestine-kidney axis injury. Additionally, activating the NRF2/KEAP1 signaling pathway promotes the function of biological antioxidant system.

**Supplementary Information:**

The online version contains supplementary material available at 10.1186/s40104-026-01382-2.

## Introduction

Suboptimal environmental conditions can induce feed mold contamination. Prolonged feeding of moldy feed increases the risk of oxidative stress and endoplasmic reticulum stress (ERS) [1]. Oxidative stress and ERS can further induce inflammatory damage to the intestine and kidney [2, 3]. Exploring natural antioxidant feed additives has become pivotal for ensuring the healthy growth of animals.

The intestine and kidney are crucial components of the gut-kidney axis. On the one hand, aflatoxin B_1_ (AFB_1_) disrupts intestinal flora homeostasis [4], leading to increased intestinal permeability, bacterial translocation, and the entry of toxic metabolites into the circulatory system [5], exacerbating renal inflammation. On the other hand, AFB_1_ induces renal oxidative stress, resulting in impairing renal function, leading to renal injury [6], which in turn disrupts the composition and function of the intestinal microbiota [1]. Therefore, an imbalance of the gut-kidney axis can cause intestinal and renal damage, reduce the level of digestive metabolism, and impair the systemic immune system of animals [7].

Mycotoxins are produced by mildewed feed, and AFB_1_ is the most toxic, difficult to remove, and widespread among them [8]. AFB_1_ is 21 times more toxic than zearalenone and 31 times more toxic than deoxynivalenol, making it the strongest class of toxic chemicals in the known class [9]. The liver is the primary organ affected by the toxicity of AFB_1_, but studies on the effects of AFB_1_ on kidney injury are important [10]. Kidney injury and intestinal barrier damage are caused by AFB_1_ via apoptosis, oxidative stress, and inflammation [11, 12]. Activation of the transcription factor 6 (ATF6)/glucose-regulated protein 78 kDa (GRP78) and interleukin 1 beta (IL-1β)/nuclear factor of kappa light polypeptide gene enhancer in B cells (NF-κB) signaling pathways is upregulated to reveal kidney injury induced by AFB_1_ [13, 14]. Inflammatory injury is triggered through signaling pathways such as the PI3K/MAPK, ROS-Erk, or NF-κB pathways [10, 15]. Therefore, intestinal and kidney injuries are the result of various biological processes and the regulation of multiple pathways. AFB_1_-induced ERS and the intestine-kidney axis are involved, providing a theoretical basis for experimental studies in ruminant model applications.

Curcumin (CUR) is a natural polyphenolic compound that contains bioactive substances with various functions, such as antioxidant, anti-inflammatory, and antitumor effects [3, 16]. The application of CUR in sheep has focused on the enhancement of reproductive performance [17], antioxidant capacity [18], and immune function [19]. In rat studies, CUR activated the NRF2/KEAP1 signaling pathway by strengthening the function of the intestinal barrier, improving antioxidant enzyme activities, and reducing the expression of inflammatory factors [20, 21]. It regulates the balance of intestinal flora, promotes the growth of beneficial bacteria, and reduces the abundance of harmful bacteria in rat [22]. Similarly, CUR treatment can attenuate the effects of AFB_1_-induced apoptosis on rat kidney injury caused by ERS and inflammation [11, 23]. However, the mechanisms how it alleviates the damage to the gut-kidney axis in sheep and promotes health are still unclear and require further research.

Dorper–Han crossbred sheep are a meat-type ovine breed derived from crossing Small-tailed Han sheep (dams) with Dorper sheep (sires). Characterized by robust adaptability and excellent stress resistance, this breed is widely farmed in China [24]. Thus, it was selected as the experimental model for AFB_1_ challenge trials.

CUR has been studied in poultry and piglets, but the mechanism through which CUR affects AFB_1_-induced intestinal and kidney function injury is unknown in sheep. Therefore, in this study, the mechanism through which CUR alleviates AFB_1_-induced intestine-kidney axis damage in sheep was investigated. These findings provide a theoretical basis for expanding the scope of CUR applications.

## Materials and methods

### Animal management and experimental procedure

Eighteen male Dorper × Small-tailed Han crossed sheep, aged 4 months and with similar body weights (29.57 ± 0.91 kg), were randomly divided into three groups (*n* = 6). The CON group was fed the total mixed ration (TMR); additionally, the AFB_1_ group received 500 μg/kg dry matter (DM) of AFB_1_ in TMR [8] and the AFB_1__CUR group received 500 μg/kg DM of AFB_1_ and 800 mg/kg DM of CUR in TMR [25]. AFB_1_ (A96590, purity ≥ 98%) was obtained from Shanghai Acmec Biochemical Co., Ltd. (Shanghai, China). CUR (S31628, purity ≥ 98%) was purchased from Shanghai Yuanye Bio-Technology Co., Ltd. (Shanghai, China). There were 21 d in the entire experimental period, and the sheep were fed the TMR diet. The TMR nutrient composition is based on the NRC (2007) guidelines [26] (Additional file 1: Table S1). All experimental sheep were housed in the same barn, with two animals per pen. They were fed twice daily at 06:00 and 18:00, respectively, with free access to feed and water. The temperature and humidity of the housing environment were maintained at optimal levels.

### Sample collection

The blood of each sheep was collected via the jugular vein in heparin sodium anticoagulant blood vessels after sacrificed and immediately centrifuged at 2,000 × *g* for 15 min, after which the upper serum sample was collected for subsequent serum index measurement; the sample was immediately sealed and frozen at −80 °C to avoid repeated freeze‒thaw cycles. Firstly, the remaining contents in the jejunum were rinsed with saline, and three continuous segments were cut from the middle of the jejunum on an ice box for H&E staining, kits, and molecular experiments. One-third of the H&E-stained jejunal segments were fixed in 4% paraformaldehyde. The remaining 2/3 of the jejunal segments were stored at −80 °C for subsequent kits and molecular experiments. A portion of the kidney was treated as the jejunum and the other portion of the kidney was quickly frozen in liquid nitrogen and stored at −80 °C for transcriptome sequencing analysis. The cecal wall was cut longitudinally, the contents were collected directly into a sterile centrifuge tube, and the sample was immediately sealed and frozen at −80 °C to avoid repeated freeze‒thaw cycles. The cecum contents were collected and stored at −80 °C for 16S rRNA sequencing analysis.

### Hematoxylin and eosin staining analysis

Kidney (renal cortex) and jejunum tissue samples (*n* = 6) were collected and stored for more than 24 h; then, the tissue blocks were dehydrated and cleared, embedded in paraffin, cut into 5 μm thick sections on a microtome, pasted on slides and dried [14]. The intestinal villus height and crypt depth were photographed and analyzed using slide viewer software (Slide Viewer 2.5; 3D HISTECH, Hungary).

### Antioxidant and kidney function index analysis

Commercial kits were used to measure the levels of hydrogen peroxide (H_2_O_2_; BC3595), malondialdehyde (MDA; BC6415), glutathione (GSH; BC1175) and the activities of glutathione S-transferase (GST; BC0355), catalase (CAT; BC0205) and superoxide dismutase (SOD; BC5165), following the manufacturer's instructions (Beijing Solarbio Technology Co., Ltd., China). Serum levels of urea nitrogen (BUN; C013-2-1), creatinine (CRE; C011-2-1) and uric acid (UA; C012-2-1) were assayed using commercial kits according to the manufacturer's instructions (Nanjing Jiancheng Bioengineering Institute, China).

### 16S rRNA sequencing analysis

Total bacterial genomic DNA was extracted from cecum content samples by using TIANamp fecal DNA Kits (Tiangen Biotech, Beijing, China). The 16S rDNA gene's V4 region was amplified by using the primer pairs 515F (5’-GTGCCAGCMGCCGCGGTAA-3’) and 806R (5’-GGACTACHVGGGTWTCTAAT-3’) and TruSeq® DNA PCR-Free Sample Preparation Kit (Illumina, USA). Sequencing libraries were generated and index codes were added on the Illumina NovaSeq platform [27]. The quality of the libraries was then evaluated using a Qubit® 2.0 Fluorometer (Thermo Fisher Scientific, Carlsbad, CA, USA) and an Agilent Bioanalyzer 2100 system. The alpha diversity was analyzed by Tukey's test and Kruskal-Wallis H test. The beta diversity was performed based on unweighted UniFrac distances. The species with significant inter-group differences were screened via the metagenomeSeq method at *P* < 0.05. The linear discriminant analysis (LDA) was performed using the linear discriminant analysis effect size software and Tax4Fun (v0.3.1) was used for functional prediction. All data visualization was implemented using R software.

### Transcriptome sequencing analysis

In accordance with the manufacturer's instructions, the total renal RNA was isolated and purified using TRIzol (Thermo Fisher, 15596018). The concentration and purity of total RNA were controlled via a NanoDrop ND-1000 (NanoDrop, Wilmington, DE, USA), and the integrity of the RNA was detected by a Bioanalyzer 2100 (Agilent, CA, USA). A concentration > 50 ng/μL, an RNA integrity number (RIN) > 7.0, and the total RNA > 1 μg were used for the subsequent experiments. Finally, double-ended sequencing was carried out using an Illumina Novaseq™ 6000 (LC Bio Technology Co., Ltd., Hangzhou, China) according to standard methods, and the sequencing mode was PE150. Reads containing adapters, more than 5% unknown nucleotides or more than 20% low-quality bases were removed to obtain highly clean data. Afterward, the clean data were compared with their paired genomes for subsequent sequencing analysis. This trial was based on the DESEQ2 algorithm (1.22.2/3.22.5); the screening conditions were log(FC) > 0.5, FC value > 1.5, and *P* < 0.05, and the comparison rate of the genome-specific data using HISAT2 software (2.2.1) was > 90%, indicating that the data were credible. Gene Ontology (GO) and Kyoto Encyclopedia of Genes and Genomes (KEGG) pathway analyses were performed using Python software, and R software version 3.6 was used for statistical analysis and to construct diagrams [27].

### RNA isolation and RT‑qPCR

Total RNA from the jejunum and kidney was extracted with TRIzol (RE703, Genesand, Beijing, China). The concentration and purity of 1 μL of RNA were measured three times per sample using a nanophotometer (NanoDrop 2000; Thermo Fisher Scientific, USA), where RNA sample purity ratios between 1.8 and 2.1 at 260/280 nm were available for further analysis. RNA reverse transcription reactions were performed using a TransScript^®^ Uni All-in-One First-Strand cDNA Synthesis SuperMix for qPCR Kit (AU341; TransGenes Biotech, Beijing, China) according to the instructions. Next, the GS AntiQ qPCR SYBR Green Fast Mix (Universal) (SQ410; Genesand Biotech, Beijing, China) was used to perform RT‑qPCR on an ABI Prism 7500 system (Applied Biosystems, Foster City, CA, USA). The primers were designed by NCBI Primer-BLAST tool and synthesized by Sangon Biotech (Shanghai, China). The 20-μL RT-qPCR volume was composed of 10 μL of 2xGS AntiQ qPCR SYBR Fast Mix (Universal), 7.2 μL of RNase-free water, 2 μL of sample cDNA and 0.4 μL of F and R primers (10 μmol/L) under the following reaction conditions: 95 °C for 30 s predenature, 95 °C for 10 s denature, and 60 °C for 30 s annealing for 44 cycles. The relative expression of target genes was calculated using the 2^−ΔΔCt^ method, and β-actin was used as the reference gene. All primers are listed in Additional file 1: Table S2.

### Western blotting analysis

The jejunum and kidney samples were fully cleaved with 1 mL of RIPA lysis buffer in a 1.5-mL tube (R0010, Solarbio, Beijing, China) and centrifuged at 13,000 × *g* and 4 °C for 15 min to obtain the supernatant for subsequent analysis. The total protein concentration was measured with an Enhanced BCA Protein Assay Kit (P0010S; Beyotime Biotechnology, Shanghai, China). The 12.5% and 10% SDS-PAGE gels (MA0388; Meilun, Dalian, China) were separated by electrophoresis in accordance with different molecular weights, and the target protein was transferred to a PVDF membrane (IPVH00010; Millipore, Burlington, USA). Afterward, the PVDF membrane was washed with 1 × TBST solution, blocked with quick closure liquid (PS108P; Epizyme, Shanghai, China), and incubated with primary antibodies and secondary antibodies (Additional file 1: Table S3). After the incubation was complete, the membrane was washed with 1 × TBST three times for 15 min each. Finally, a UVItec Gel imaging system was used to obtain the relevant bands, and the ECL reagent was added.

### Statistical analysis

The SPSS 26.0 software was used for statistical analysis and the experimental data were presented as mean ± standard deviation (SD). Multiple group comparisons were analyzed by one-way analysis of variance (ANOVA) and followed by Tukey's post hoc test, with significance levels indicated as follows: ^*^P < 0.05, ^**^P < 0.01; ns, not significant. GraphPad Prism 10.1 software was used for graphing. Correlation analysis between differential flora and serum indicators was carried out by Spearman correlation analysis, and cluster analysis was performed on the basis of the complete algorithm; *P* < 0.05 indicated that there was a significant difference between the flora and the index.

## Results

### Effects of CUR and AFB_1_ induction on the jejunal phenotype and antioxidant capacity

**Fig. 1:**
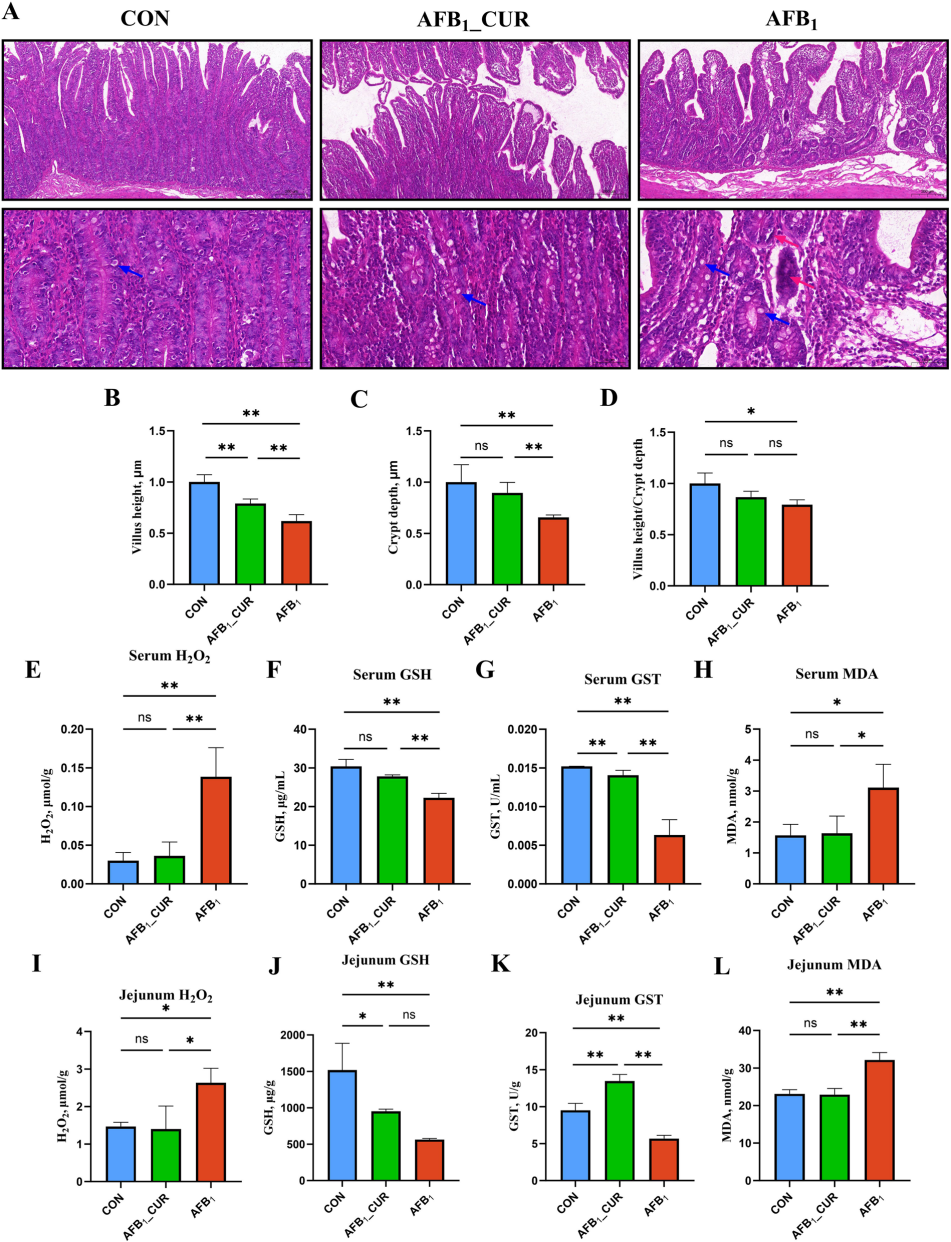
Jejunal apparent indicators (*n* = 6). **A** HE stained sections of jejunum (200 μm and 50 μm; the blue arrows indicate goblet cells, and the red arrows indicate lymphocytes.). **B**–**D** The relative villous height, relative crypt depth, and villus height/crypt rate of the jejunum. **E**–**H** Serum hydrogen peroxide (H_2_O_2_) content, glutathione (GSH) content, glutathione S-transferase activity (GST) activity, and malondialdehyde (MDA) content. **I**–**L** H_2_O_2 _content, GSH content, GST activity, and MDA content on jejunal tissues. CON: Sheep fed with basic feed. AFB_1__CUR: Sheep fed with 500 μg/kg DM of AFB_1_ and 800 mg/kg DM CUR. AFB_1_: Sheep fed with 500 μg/kg DM of AFB_1_. All data are presented as mean ± SD. ^*^P < 0.05, ^**^P < 0.01; ns, not significant

Compared with those in the CON group, the jejunal mucosal damage in the AFB_1_ group showed disordered and sparse villus arrangement, and reduced relative villus height (*P* < 0.05), crypt depth (*P* < 0.01) and villus crypt rate (VCR; *P* < 0.05). Also, goblet cells decreased, lymphocytes increased, and connective tissue became loose, but CUR alleviated the mucosal damage caused by AFB_1_ (Fig. 1A–D). The serum H_2_O_2_ content (*P* < 0.01) and MDA content (*P* < 0.05) in the AFB_1_ group were significantly increased, but were significantly decreased by CUR (Fig. 1E and H). The antioxidant capacity decreased because of the addition of AFB_1_, and the serum content of GSH and the activity of GST (*P* < 0.01) was significantly decreased by AFB_1_, but the opposite occurred in the CUR group (*P* < 0.01; Fig. 1F and G). The trends in jejunal tissue were the same as that in serum. Compared with the CON group, the H_2_O_2_ (*P* < 0.01) and MDA contents (*P* < 0.05) in the AFB_1_ group significantly increased, and the content of GSH and the activity of GST (*P* < 0.01) significantly decreased, but CUR reversed these trends (Fig. 1I–L).

### CUR regulates AFB_1_-induced gut microbiota structure disorder

**Fig. 2:**
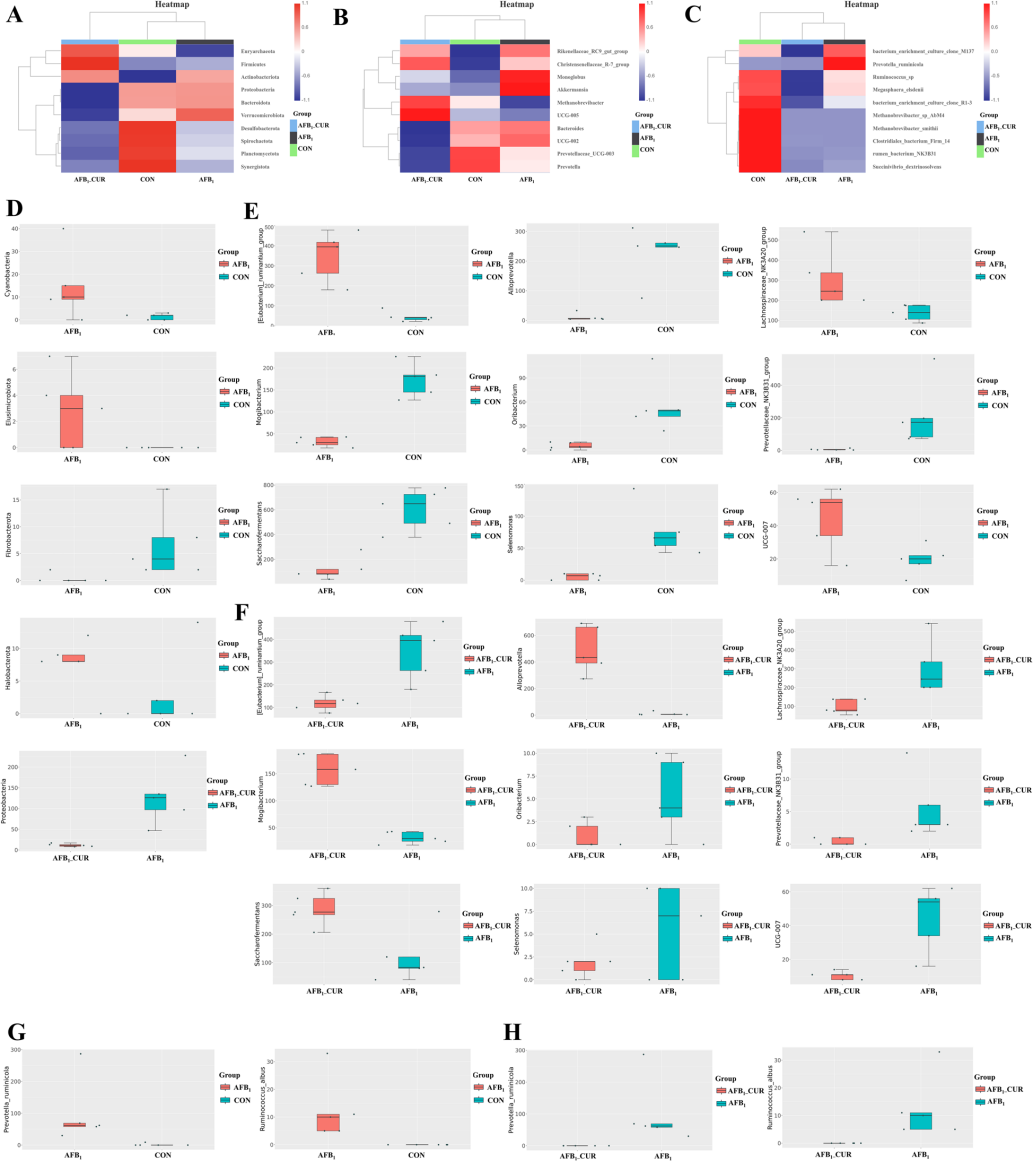
Differential flora at the phylum, genus, and species levels (*n* = 5). **A**–**C** Relative heat map of the top 10 microbial abundance in the cecum at the phylum, genus and species levels. **D** Cecal differential microbes at the phylum level (*P* < 0.05). **E** Cecal differential microorganisms at the genus level (CON group vs. AFB_1_ group; *P* < 0.05). **F** Differential microorganisms in the cecum at the genus level (AFB_1_ group vs. AFB_1__CUR group; *P* < 0.05). **G** Cecal differential microorganisms at the species level (CON group vs. AFB_1_ group; *P* < 0.05). **H** Cecal differential microorganisms at the species level (AFB_1_ group vs. AFB_1__CUR group; *P* < 0.05)

At the phylum level, microorganisms with high cecal abundance included Euryarchaeota, Firmicutes, Actinobacteria, Proteobacteria, and Bacteroidota (Fig. 2A). At the genus level, the following microorganisms had high cecal abundance: *Rikenellaceae*_*RC9*_*gut*_*group*, *Monoglobus*, *UCG*–*005*, *Bacteroides*, and *Prevotella* (Fig. 2B). At the species level, the following microorganisms had high cecal abundance: *Bacterium*_enrichment_*culture*_*clone*_*M137*, *Prevotella ruminicola*, and *Ruminococcus* sp. (Fig. 2C).

At the phylum level, compared with CON group, the microbial abundance (*P* < 0.05) of Cyanobacteria, Elusimicrobiota, and Halobacterota in the AFB_1_ group significantly increased, and the abundance (*P* < 0.05) of Fibrobacterota significantly decreased. The abundance of the pathogenic bacteria (*P* < 0.05) of Proteobacteria in the AFB_1__CUR group was significantly reduced (Fig. 2D).

At the genus level, the microbial abundance (*P* < 0.05) of [*Eubacterium*]_*ruminantium*_*group*, *Lachnospiraceae*_*NK3A20*_*group*, and *UCG*–*007* in the AFB_1_ group significantly increased, and the abundance (*P* < 0.05) of the microorganisms *Alloprevotella*, *Mogibacterium*, *Oribacterium*, *Prevotellaceae*_*NK3B31*_*group*, *Saccharofermentans*, and *Selenomonas* in the AFB_1_ group significantly decreased (Fig. 2E). Among them, the bacterial abundance (*P* < 0.05) of *Alloprevotella*, *Mogibacterium*, and *Saccharofermentans* increased, and the abundance of [*Eubacterium*]_*ruminantium*_*group*, *Oribacterium, Selenomonas*, *Lachnospiraceae*_*NK3A20*_*group*, *Prevotellaceae*_*NK3B31*_*group* and *UCG*–*007* (*P* < 0.05) significantly decreased by CUR (Fig. 2F).

At the species level, the abundance of *Prevotella* *ruminicola* and *Ruminococcus* *albus* significantly increased in the AFB_1_ group (*P* < 0.05), and their abundance significantly decreased in the AFB_1__CUR group compared with the AFB_1_ group (*P* < 0.05; Fig. 2G–H). In conclusion, the abundance of some pathogenic bacteria increased in response to AFB_1_ treatment, and this effect was alleviated by the addition of CUR.

### CUR regulates AFB_1_-induced changes in gut microbiota function

**Fig. 3:**
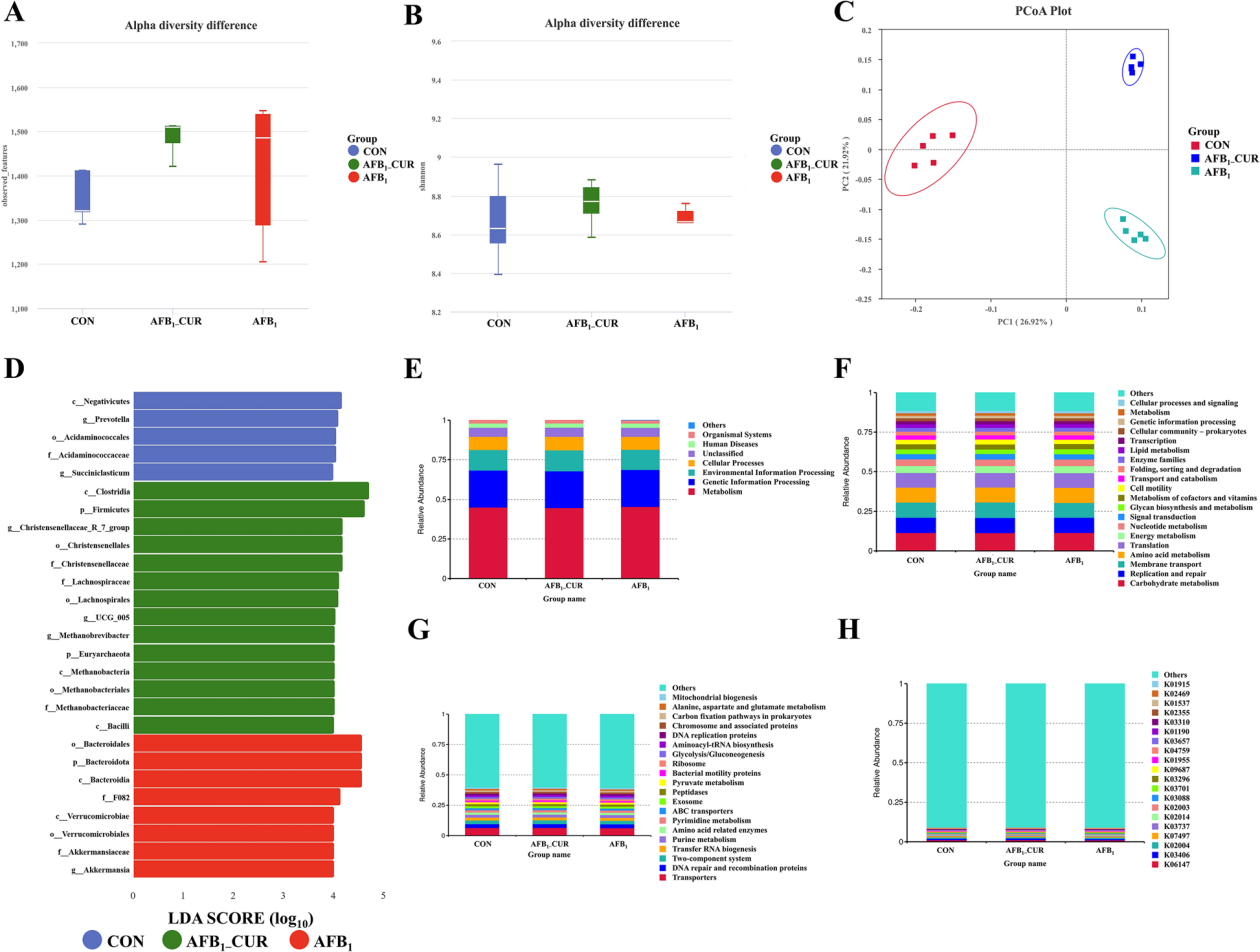
Cecal microbial diversity and prediction of gut microbiota function (*n* = 5). **A** and **B** The α diversity of the observed features index (flora abundance) and Shannon index (flora diversity) in the cecal microbial (*P* > 0.05), the white horizontal line is the average value size. **C** Principal co-ordinates analysis (PCoA) analysis of cecal microbial β diversity. **D** Linear discriminant analysis in cecal microorganisms. **E**–**H** Functional analysis of cecal microorganisms with top 20 Kyoto Encyclopedia of Genes and Genomes (KEGG) enrichment pathways level1, level2, level3 and level4

As shown in Fig. 3, the observed α diversity index and Shannon index showed no significant difference among the three groups. However, β diversity analysis revealed that the samples in the CON group, AFB_1_ group and AFB_1__CUR group had good discreteness, indicating that the components could be separated and that the data were valid (Fig. 3C). Linear discriminant analysis (LDA) can predict the potential markers that separate each experimental group, the results revealed 27 potential biomarkers that could effectively separate the biomarkers of each group. At the genus level, CON group biomarkers *Prevotella* and *Succiniclasticum*; the AFB_1__CUR group biomarkers *Christensenellaceae*_*R7*_*group, UCG*–*005* and *Methanobrevibacter*, and the AFB_1_ group biomarker *Akkermansia* (Fig. 3D). Microbial species are usually associated with biological functions, and prediction of bacterial community functions could be anticipated with Tax4Fun function prediction. The KEGG level 3 results revealed that pathways were enriched mainly in two-component systems, amino acid-related enzymes, peptidases, and alanine, aspartate and glutamate metabolism (Fig. 3E–G). At the KEGG level 4, key pathway-related genes included pyruvate-ferredoxin/flavonoid toxin oxidoreductase, antitoxin, ferrous transporter, β-galactosidase, Ca^2+^ transporter ATPase, and glutamine synthase (Fig. 3H).

### Effects of CUR and AFB_1_ induction on jejunal barrier and apoptotic gene expression

**Fig. 4:**
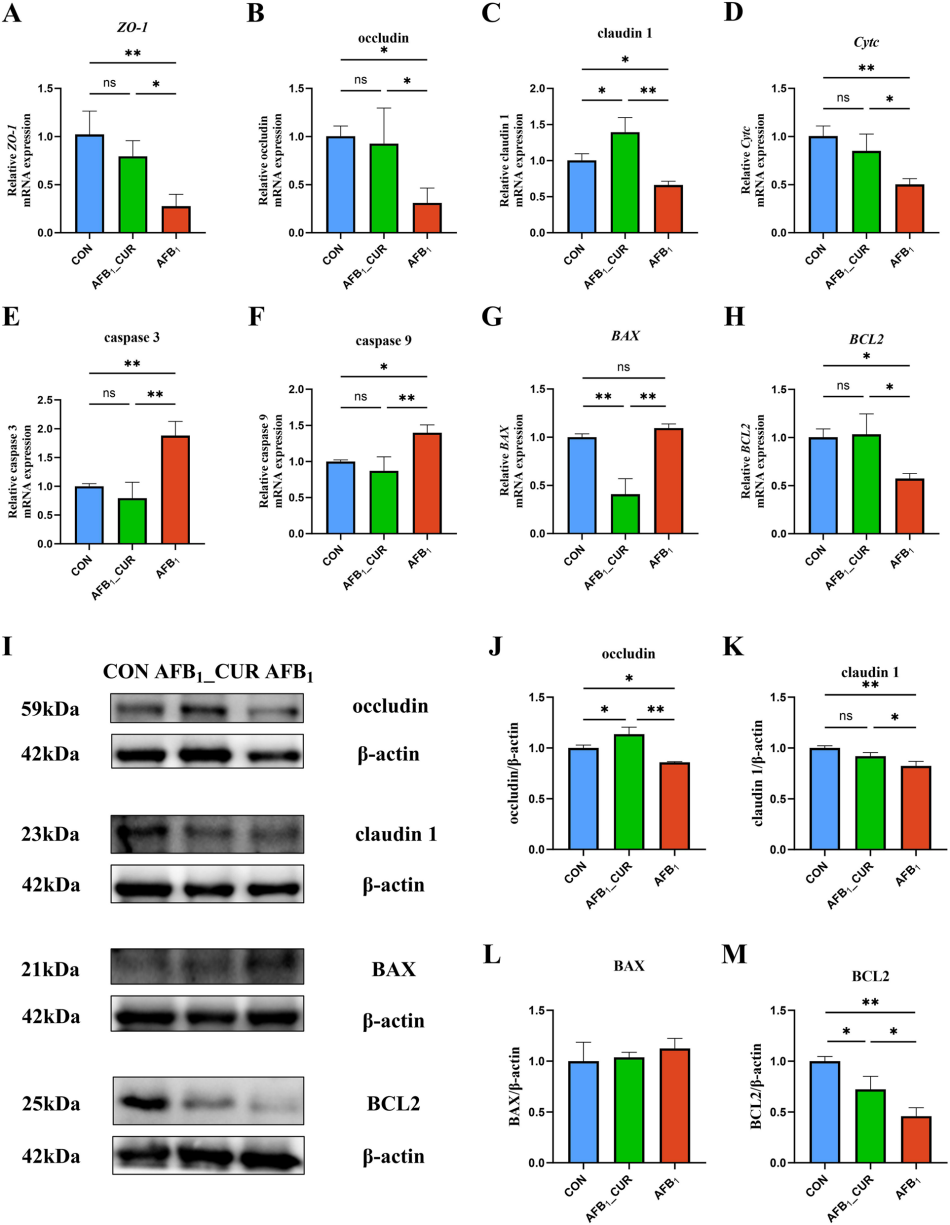
Jejunal intestinal barrier and apoptosis related genes’ mRNA (*n* = 6) and protein expression (*n* = 3). **A**–**C** Relative mRNA expression of jejunal intestinal barrier-related genes. *ZO-1*, tight junction protein 1. **D**–**H** Relative mRNA expression of jejunal apoptosis-related genes. *Cytc*, cytochrome C; *BAX*, BCL2 associated X; *BCL2*, BCL2 apoptosis regulator. **I**–**M** Jejunal intestinal barrier and apoptotic protein expression. All data are presented as mean ± SD. ^*^P < 0.05, ^**^P < 0.01; ns, not significant

AFB_1_ inhibited jejunal intestinal barrier-related genes, and CUR alleviated the harmful effect of AFB_1_. The relative mRNA expression of tight junction protein 1 (*ZO-1*; *P* < 0.01) and occludin and claudin 1 (*P* < 0.05) in the AFB_1_ group significantly decreased, and intestinal barrier-related gene expression increased in response to CUR treatment (Fig. 4A–C). The jejunum in the AFB_1_ group was in an apoptotic state, and CUR alleviated apoptosis. In the AFB_1_ group, the relative mRNA expression of cytochrome C (*Cytc*; *P* < 0.01) and BCL2 apoptosis regulator (*BCL2*; *P* < 0.05) was significantly lower, and the relative expression of caspase 3 (*P* < 0.01) and caspase 9 (*P* < 0.05) was significantly greater. However, compared with AFB_1_ group, the mRNA expression of apoptosis-related genes (*P* < 0.01 or *P* < 0.05) was reversed in the CUR group, and the expression of BCL2-associated X (*BAX*; *P* < 0.01) was significantly reduced (Fig. 4D–H).

Similarly, the protein expression of occludin (*P* < 0.05) and claudin 1 (*P* < 0.01) significantly decreased by AFB_1_, and BCL2 protein expression (*P* < 0.05) was decreased. In the AFB_1__CUR group, the protein expression of occludin (*P* < 0.01) and claudin 1 (*P* < 0.05) was increased, and the expression of BCL2 (*P* < 0.05) was increased (Fig. 4I–M).

### NRF2 signaling activation in the jejunum by CUR

**Fig. 5:**
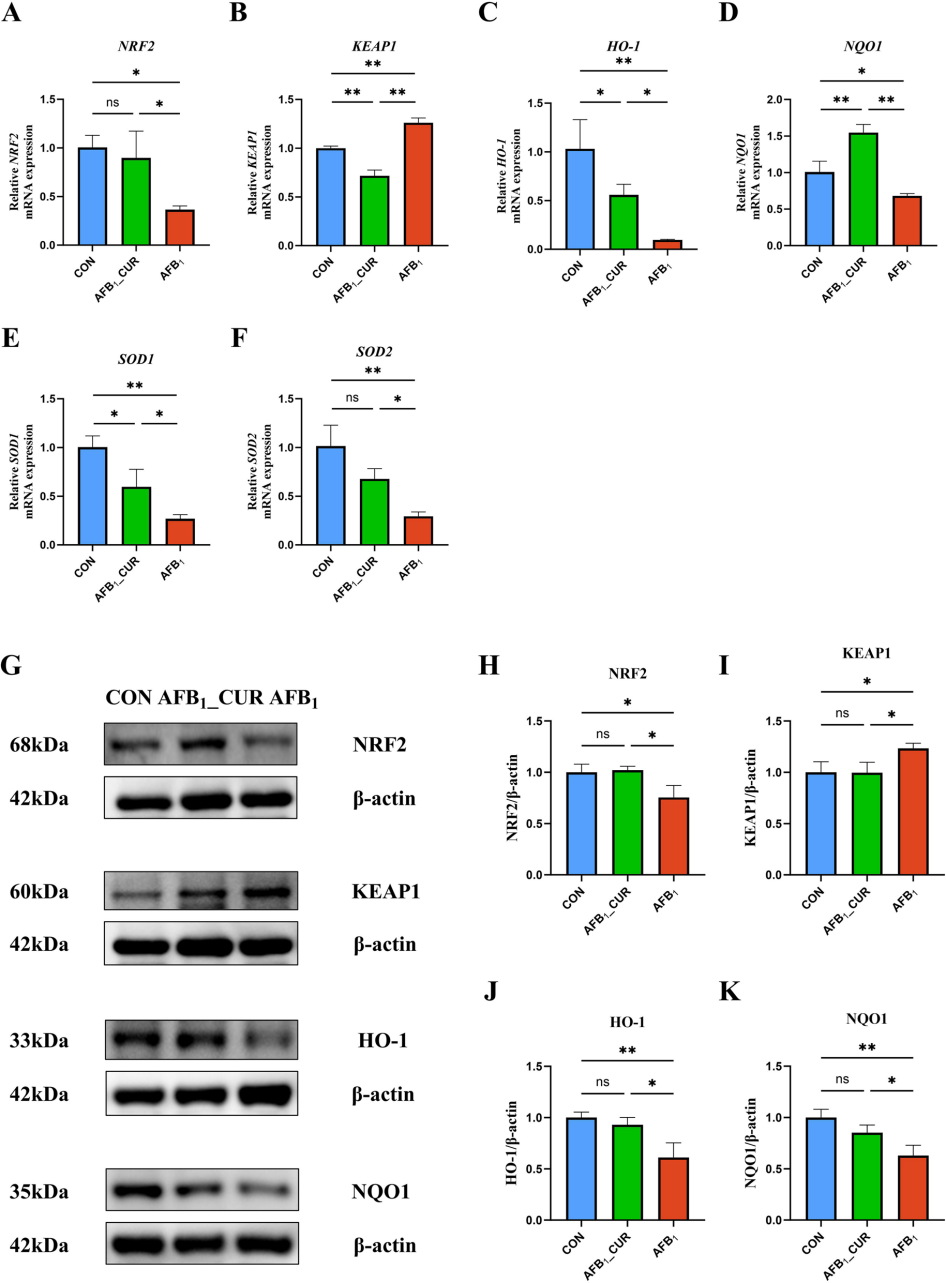
CUR regulates the mRNA (*n* = 6) and protein (*n* = 3) expression of genes related to the jejunal NRF2 signaling pathway. **A**–**F** Relative mRNA expression of NRF2 signaling pathway and downstream genes. *NRF2*, nuclear factor erythroid 2-related factor 2; *KEAP1*, Kelch like ECH associated protein 1; *HO-1*, heme oxygenase 1; *NQO1*, NAD(P)H quinone dehydrogenase 1; *SOD1*, superoxide dismutase 1; *SOD2*, superoxide dismutase 2. **G**–**K** The expression of proteins related to the jejunal NRF2 signaling pathway. All data are presented as mean ± SD. ^*^P < 0.05, ^**^P < 0.01; ns, not significant

AFB_1_ could cause jejunal oxidative stress, whereas CUR could activate the jejunal antioxidant system and resist the oxidative stress induced by AFB_1_ to a certain extent. As shown in Fig. 5, the relative mRNA expression of nuclear factor erythroid 2-related factor 2 (*NRF2*; *P* < 0.05) was decreased and the relative expression of Kelch-like ECH-associated protein 1 (*KEAP1*; *P* < 0.01) was significantly increased; the relative mRNA expression of *NRF2* signaling pathway downstream genes heme oxygenase 1 (*HO-1*; *P* < 0.01), NAD(P)H quinone dehydrogenase 1 (*NQO1*; *P* < 0.05), superoxide dismutase 1 (*SOD1*; *P* < 0.01) and superoxide dismutase 2 (*SOD2*; *P* < 0.01) in the AFB_1_ group was decreased. In contrast, mRNA expression of *NRF2* signaling pathway genes was increased (*P* < 0.01 or *P* < 0.05) and the relative expression of *KEAP1* (*P* < 0.01) was significantly decreased by CUR (Fig. 5A–F).

Western blot analysis of the NRF2 pathway was performed to verify that CUR can significantly reduce oxidative stress damage caused by AFB_1_. Compared with the CON group, the protein expression of NRF2, HO-1 and NQO1 in the AFB_1_ group was decreased (*P* < 0.01 or *P* < 0.05), and the protein expression of KEAP1 was increased (*P* < 0.05). However, the protein expression of these genes (*P* < 0.01 or *P* < 0.05) was opposite in the AFB_1__CUR group, indicating that CUR can activate the antioxidant system to resist oxidative stress damage caused by AFB_1_ (Fig. 5G–K).

### NF-κB signaling pathway suppression in the jejunum by CUR

**Fig. 6:**
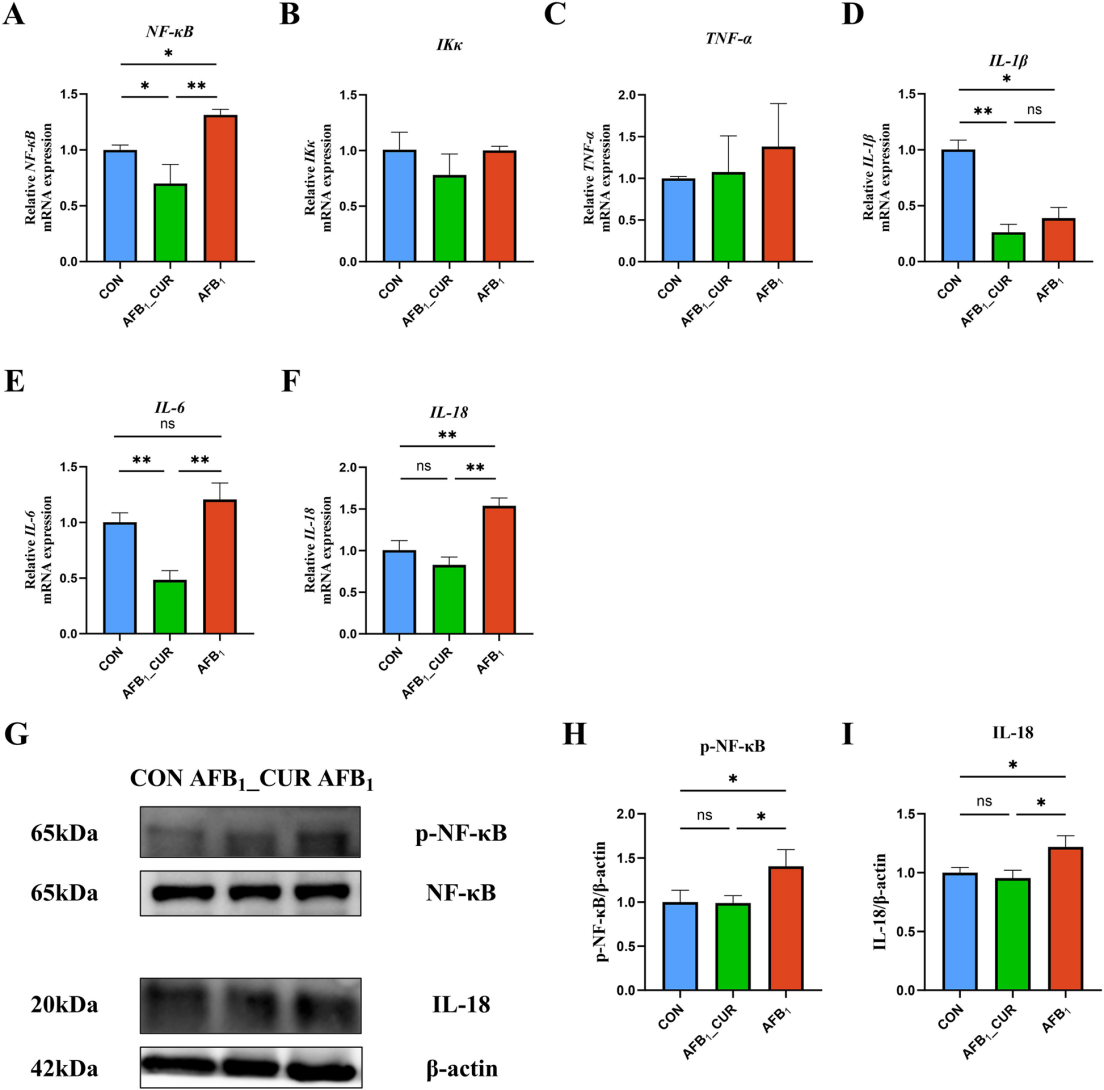
CUR regulates the mRNA (*n* = 6) and protein (*n* = 3) expression of genes related to the jejunal NF-κB signaling pathway. **A**–**F** Relative mRNA expression of jejunal NF-κB signaling pathway and inflammatory factor. *NF-κB*, nuclear factor of kappa light polypeptide gene enhancer in B cells; *IKκ*, inhibitor of nuclear factor kappa B kinase ; *TNF-α*, tumor necrosis factor α; *IL-1β*, interleukin 1 beta; *IL-6*, interleukin 6; *IL-18*, interleukin 18. **G**–**I** Expression of phosphorylated NF-κB and pro-inflammatory factor IL-18 protein in the jejunum. All data are presented as mean ± SD. ^*^P < 0.05, ^**^P < 0.01; ns, not significant

ROS is a key indicator that connects NRF2 with NF-κB, and the level of H_2_O_2_ was increased by AFB_1_ to activate the NF-κB signaling pathway and restrain the NRF2 signaling pathway to activate the antioxidant system. The relative mRNA expression levels of *NF-κB* (*P* < 0.05), *IL-1β* (*P* < 0.05) and interleukin 18 (*IL-18*; *P* < 0.01) in the AFB_1_ group were markedly greater; however, they were markedly lower in the AFB_1__CUR (*P* < 0.01; Fig. 6A–F).

The above results revealed that *IL-18* is a potential key gene that regulates the inflammatory response. Therefore, protein expression of p-NF-κB and IL-18 (*P* < 0.05) was increased by AFB_1_ but decreased by CUR (*P* < 0.05). The results showed that CUR inhibited the activation of the NF-κB signaling pathway and alleviated the inflammatory damage caused by AFB_1_ to the jejunum (Fig. 6G–I).

### Effects of CUR and AFB_1_ induction on kidney phenotype and antioxidant capacity

**Fig. 7:**
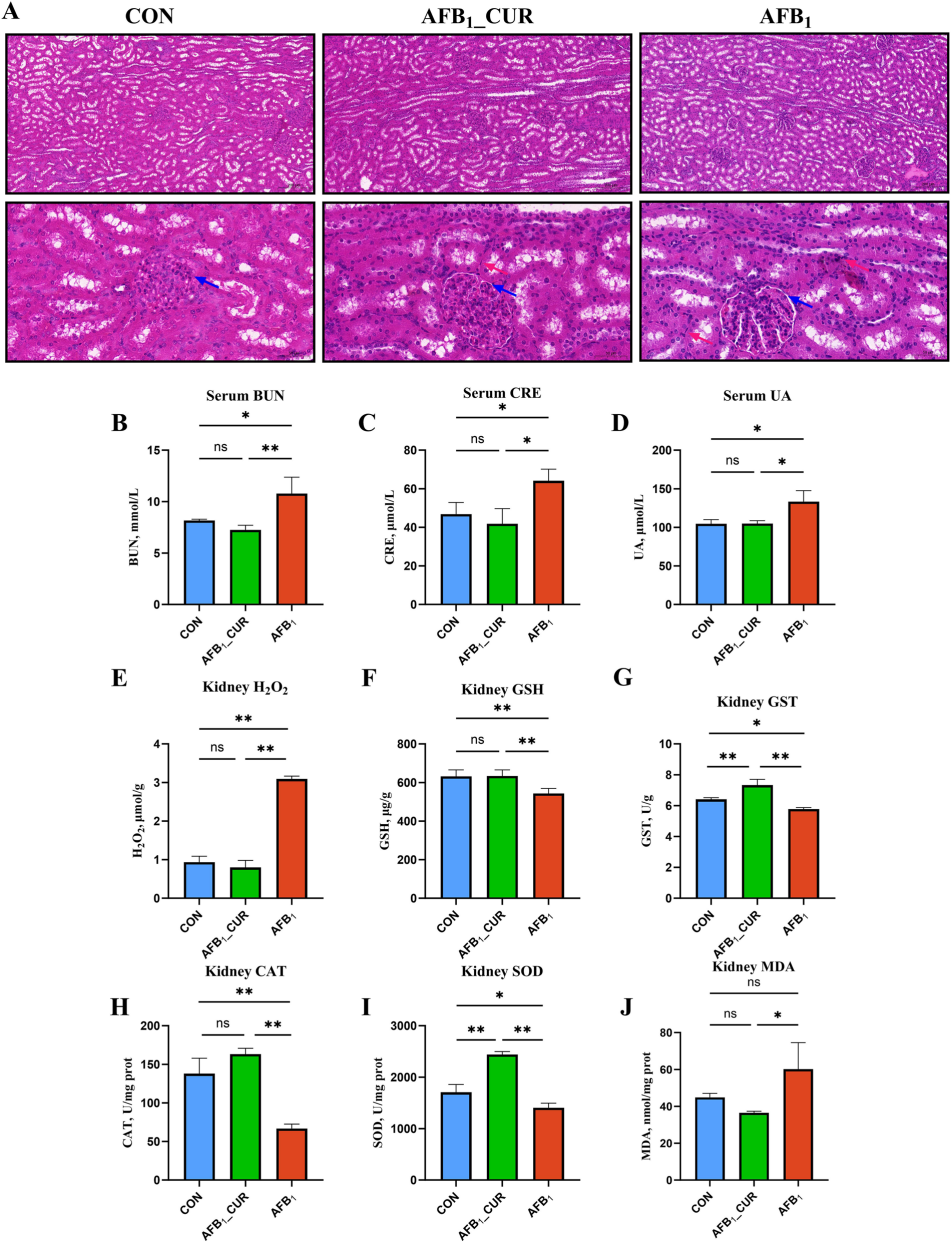
Kidney apparent indicators (*n* = 6). **A** HE stained sections of kidneys (200 μm and 50 μm, the blue arrows indicate the capsular space of renal corpuscle, and the red arrows indicate inflammatory cells). **B**–**D** Serum levels of BUN, CRE and UA. **E**–**G** H_2_O_2_ content, GSH content and GST activity in kidney tissues. **H**–**I** CAT activity, SOD activity and MDA content in kidney tissues. All data are presented as mean ± SD. ^*^P < 0.05, ^**^P < 0.01; ns, not significant

The results of the HE staining revealed glomerular shrinkage in the AFB_1_ group; vacuolization was observed in the Bowman’s capsule space; eosinophilic granular proteinaceous substances were visible in the capsule space, and renal tubules showed irregular morphology with inflammatory cell infiltration (Fig. 7A). BUN, CRE and UA are recognized as renal function indices for assessing renal damage. The serum BUN content, CRE content and UA content (*P* < 0.05) increased in the AFB_1_ group; in contrast, the kidney indicators decreased in the AFB_1__CUR group (*P* < 0.01 or *P* < 0.05; Fig. 7B–D). H_2_O_2_ content can reflect redox levels and is a key marker of oxidative stress reactions. Compared with the CON group, the kidney H_2_O_2_ content (*P* < 0.01) in the AFB_1_ group increased but was significantly lower in the AFB_1__CUR group (*P* < 0.01; Fig. 7E). However, the GSH content and the activities of CAT, GST and SOD were reduced by AFB_1_ (*P* < 0.01 or *P* < 0.05), and CUR treatment eased this reduction and decreased the MDA content (*P* < 0.01 or *P* < 0.05; Fig. 7F–J).

### Effects of CUR and AFB_1_ induction on kidney transcriptome differential gene expression

**Fig. 8:**
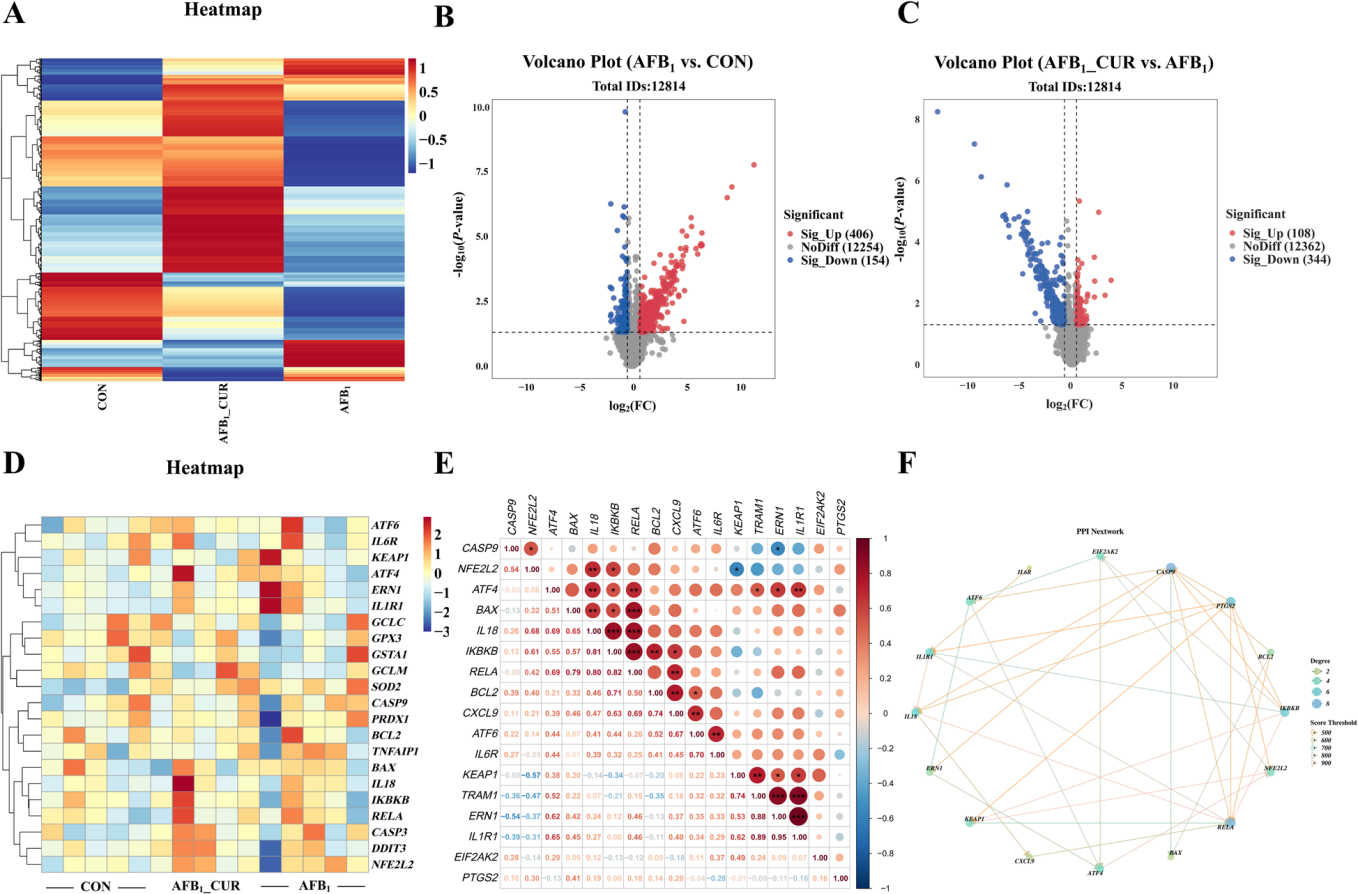
Kidney transcriptome differential gene analysis (*n* = 5). **A** Heatmap of differentially expressed genes in kidney. **B** Volcanic plot of differentially expressed genes in CON group and AFB_1_ group (red dots indicate rising genes, blue dots indicate down-regulated genes, and gray dots indicate genes with insignificant differences). **C** Volcano plot of differentially expressed genes in AFB_1_ group and AFB_1__CUR group. **D** Heatmap of individual differential gene expression, red up-regulated and blue down-regulated. *GSTA1*, glutathione S-transferase alpha 1; *PRDX1*, peroxiredoxin 1; *EIF2AK2*, eukaryotic translation initiation factor 2 alpha kinase 2; *ATF4*, activating transcription factor 4; *RELA*, RELA proto-oncogene; *CXCL9*, C-X-C motif chemokine ligand 9; *IL6R*, interleukin 6 receptor; *CASP9,* caspase 9. **E** Correlation coefficients of different differential gene expressions (^*^P < 0.05, ^**^P < 0.01, ^***^P < 0.001). **F** Network diagram of different genes and proteins (dot size indicates the number of related objects, line/line color represents the strength of correlation)

The gene expression profiles of the CON group, the AFB_1__CUR group and the AFB_1_ group were compared, and the gene expression of the AFB_1__CUR group was essentially the same as that of the CON group (Fig. 8A). Compared with the CON group, the AFB_1_ group had 406 upregulated genes and 154 downregulated genes, and compared with the AFB_1_ group, the AFB_1__CUR group had 108 upregulated genes and 344 downregulated genes (Fig. 8B and C). In the AFB_1_ group, genes related to apoptosis, ERS and inflammatory factors were upregulated. Compared with those in the AFB_1_ group, the expression of NRF2 pathway-related genes in AFB_1__CUR, including *NRF2*, glutathione S-transferase alpha 1 (*GSTA1*) and peroxiredoxin 1 (*PRDX1*), tended to increase, but the expression of genes related to ERS and inflammatory factors tended to decrease (Fig. 8D). Eukaryotic translation initiation factor 2 alpha kinase 2 (*EIF2AK2*) and activating transcription factor 4 (*ATF4*) were significantly positively correlated with *KEAP1* (*P* < 0.01 or *P* < 0.05), *NRF2* was positively correlated with *IL-18* (*P* < 0.01) and RELA proto-oncogene (*RELA*), *IL-18* was positively correlated with *BAX* (*P* < 0.01) and *RELA* (*P* < 0.001), and *ATF6* was positively correlated with C-X-C motif chemokine ligand 9 (*CXCL9*) and interleukin 6 receptor (*IL6R*) (*P* < 0.01; Fig. 8E). The protein interaction network diagram revealed that *NRF2* was strongly correlated with *KEAP1*, *EIF2AK2* and *ATF4* and that *BAX* was strongly correlated with *BCL2* and caspase 9 (*CASP9*), that *ATF6* was strongly correlated with *EIF2AK2*, and that *RELA* was strongly correlated with *IL-18* and *IL-1R1*; thus, *NRF2* and endoplasmic reticulum stress-related genes were strongly correlated (Fig. 8F).

### Regulatory effects of CUR and AFB_1_ on differential gene expression in the kidney transcriptome

**Fig. 9:**
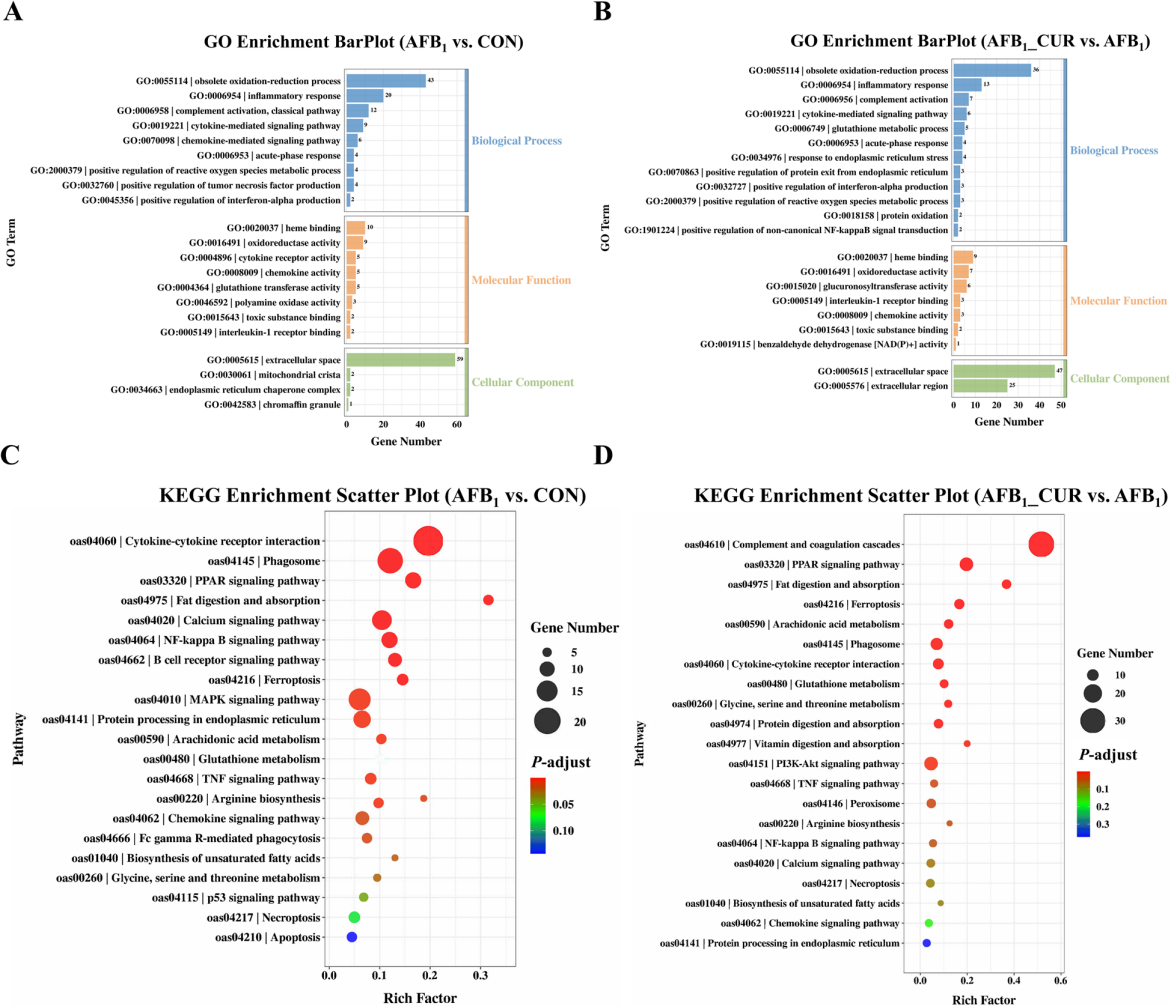
GO and KEGG enrichment analysis (*n* = 5). **A** GO enrichment barplot of CON group and AFB_1_ group (blue refers to biological process, orange refers to molecular function, green refers to cell components). **B** GO enrichment barplot of AFB_1_ and AFB_1__CUR groups. **C** Image of KEGG pathway enrichment factor in CON group and AFB_1_ group. **D** Image of KEGG pathway enrichment factor in AFB_1_ and AFB_1__CUR groups

GO enrichment histograms revealed that the differentially expressed genes in the AFB_1_ group were enriched mainly in the biological process, molecular function and cellular component terms, and the genes related to these terms were enriched mainly in the following biological processes: obsolete oxidation–reduction process, inflammatory response, chemokine-mediated signaling pathway, acute-phase response, positive regulation of reactive oxygen species metabolic process, and positive regulation of interferon-alpha production; in terms of molecular function, the genes were enriched mainly in oxidoreductase activity, glutathione transferase activity, polyamine oxidase activity, toxic substance binding, and interleukin-1 receptor binding; and the cell components were concentrated mainly in the extracellular space and endoplasmic reticulum chaperone complex (Fig. 9A). The AFB_1_ group was compared with the AFB_1__CUR group, the biological processes of the relevant genes were enriched mainly in the obsolete oxidation–reduction process, inflammatory response, glutathione metabolic process, acute-phase response, response to endoplasmic reticulum stress, positive regulation of protein exit from endoplasmic reticulum, positive regulation of interferon-alpha production and positive regulation of noncanonical NF-κB signal transduction; secondly, the molecular functions were concentrated mainly in oxidoreductase activity, glucuronosyltransferase activity, interleukin-1 receptor binding, and toxic substance binding, and benzaldehyde dehydrogenase [NAD(P)^+^] activity; thirdly, the composition of cells was concentrated mainly in the extracellular region (Fig. 9B). The results of the KEGG enrichment analysis revealed that the main pathways in the AFB_1_ group were enriched in fat digestion and absorption, calcium signaling, NF-κB signaling, ferroptosis, protein processing in the endoplasmic reticulum, glutathione metabolism, TNF signaling, arginine biosynthesis, biosynthesis of unsaturated fatty acids and apoptosis (Fig. 9C). The results of the KEGG enrichment analysis revealed that compared with the AFB_1_ group, the main pathways in the AFB_1__CUR group were enriched in fat digestion and absorption, ferroptosis, glutathione metabolism, TNF signaling pathway, arginine biosynthesis, NF-κB signaling pathway, calcium signaling pathway, unsaturated fatty acid biosynthesis and protein processing in the endoplasmic reticulum (Fig. 9D).

### CUR decreases AFB_1_-induced apoptosis-related gene expression

**Fig. 10:**
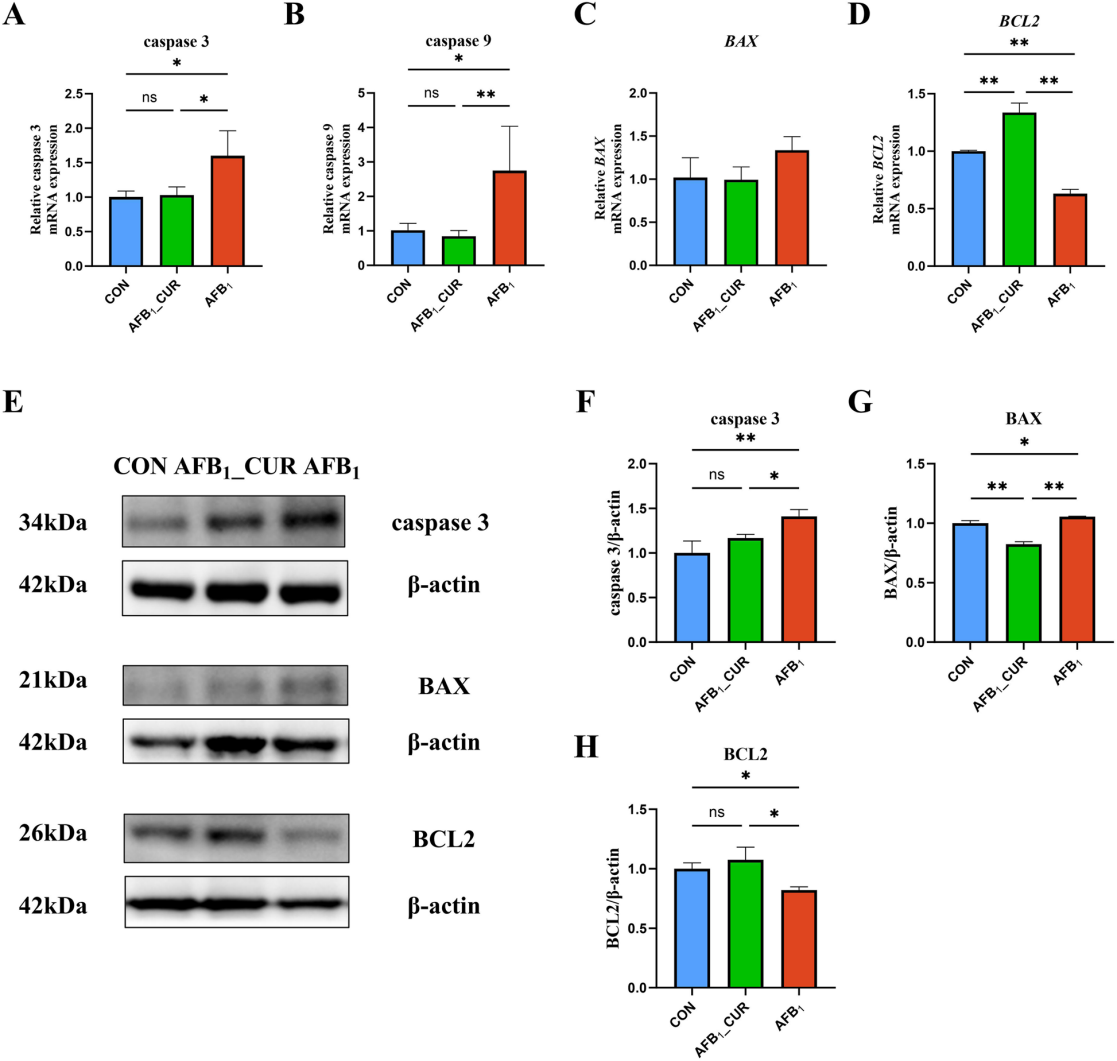
CUR regulates the mRNA (*n* = 6) and protein (*n* = 3) expression of apoptosis-related genes. **A**–**D** Relative mRNA expression of kidney apoptosis-related genes. **E**–**H** Expression of kidney apoptosis-related proteins. All data are presented as mean ± SD. ^*^P < 0.05, ^**^P < 0.01; ns, not significant

AFB_1_ can induce acute stress responses in animals, resulting in apoptosis and a series of stress responses in the kidney, ultimately resulting in kidney injury. In contrast to the CON group, the relative mRNA expression of caspase 3 (*P* < 0.05) was greater in the AFB_1_ group, and the relative expression of *BCL2* (*P* < 0.01) was significantly lower. The relative mRNA expression of caspase 9 and caspase 3 (*P* < 0.05) was lower and that of *BCL2* (*P* < 0.01) was greater in the AFB_1__CUR group (Fig. 10A–D).

Similarly, the protein expression of caspase 3 (*P* < 0.01) and BAX (*P* < 0.05) increased, and the protein expression of BCL2 (*P* < 0.05) decreased in the AFB_1_ group; in the AFB_1__CUR group, caspase 3 (*P* < 0.05) and BAX (*P* < 0.01) exhibited markedly decreased expression, while BCL2 protein expression increased (*P* < 0.05; Fig. 10E–H).

### NRF2 signaling in the kidney was activated by CUR

**Fig. 11:**
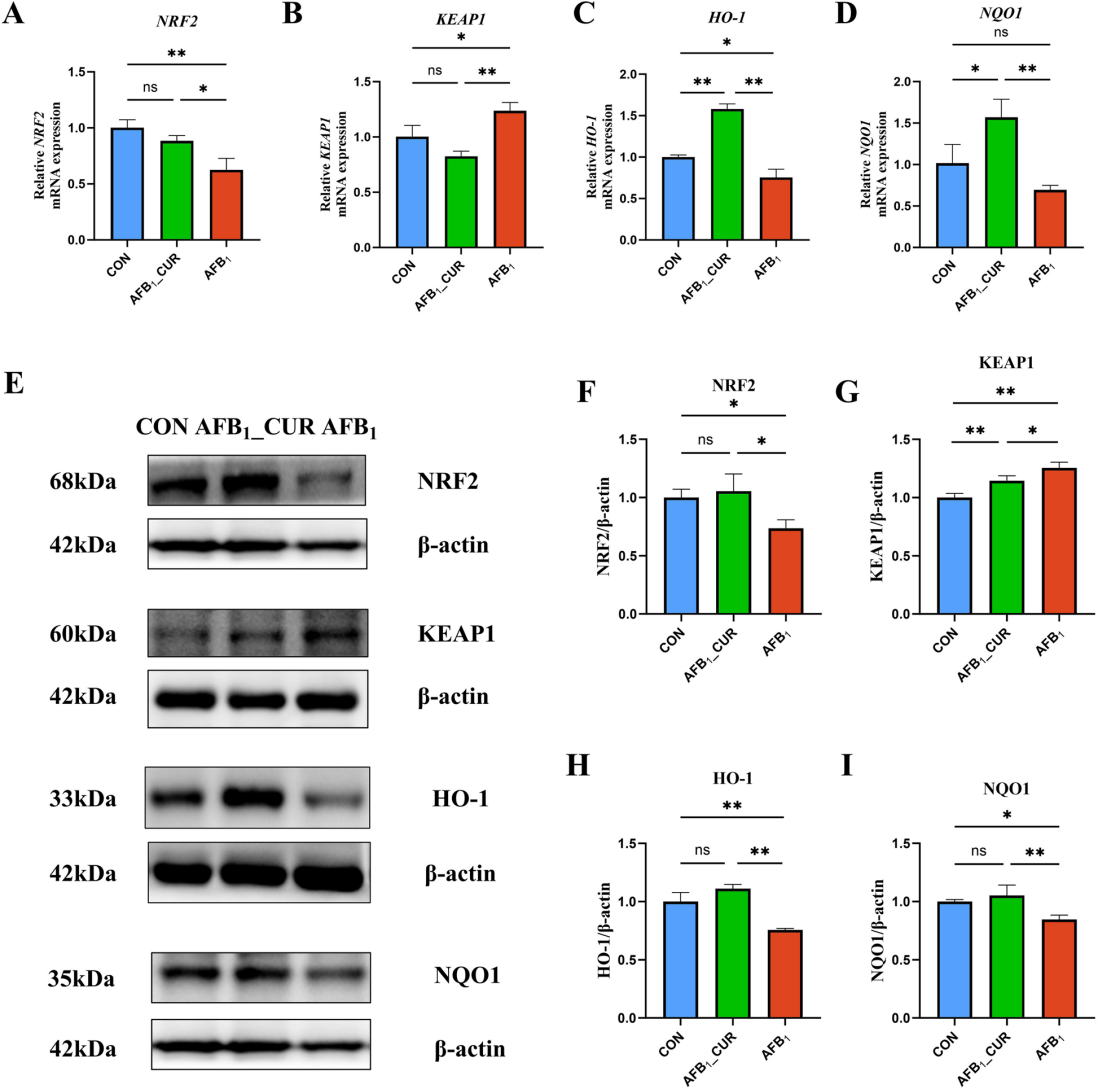
CUR regulates the mRNA (*n* = 6) and protein (*n* = 3) expression of genes related to the NRF2 signaling pathway. **A**–**D** Relative mRNA expression of NRF2 pathway-related genes in the kidney. **E**–**I** Expression of NRF2 pathway-related proteins in the kidney. All data are presented as mean ± SD. ^*^P < 0.05, ^**^P < 0.01; ns, not significant

The relative mRNA expression of *NRF2* (*P* < 0.01) and the downstream genes of *HO-1* (*P* < 0.05) and *NQO1* was inhibited by AFB_1_ treatment, and the relative mRNA expression of *KEAP1* (*P* < 0.05) was increased. In contrast, supplementation with CUR increased the expression of the *NRF2*, *HO-1* and *NQO1* genes, and the relative expression of the *KEAP1* was suppressed (*P* < 0.01 or *P* < 0.05; Fig. 11A–D).

Compared with that in the CON group, the protein expression of NRF2 (*P* < 0.05) and HO-1 (*P* < 0.01) in the AFB_1_ group was markedly lower, and the protein expression of KEAP1 was markedly greater (*P* < 0.01). However, they significantly increased (*P* < 0.01 or* P* < 0.05), and KEAP1 expression significantly decreased after CUR treatment (*P* < 0.05; Fig. 11E–I).

### ATF6/GRP78 signaling in the kidney was inhibited by CUR

**Fig. 12:**
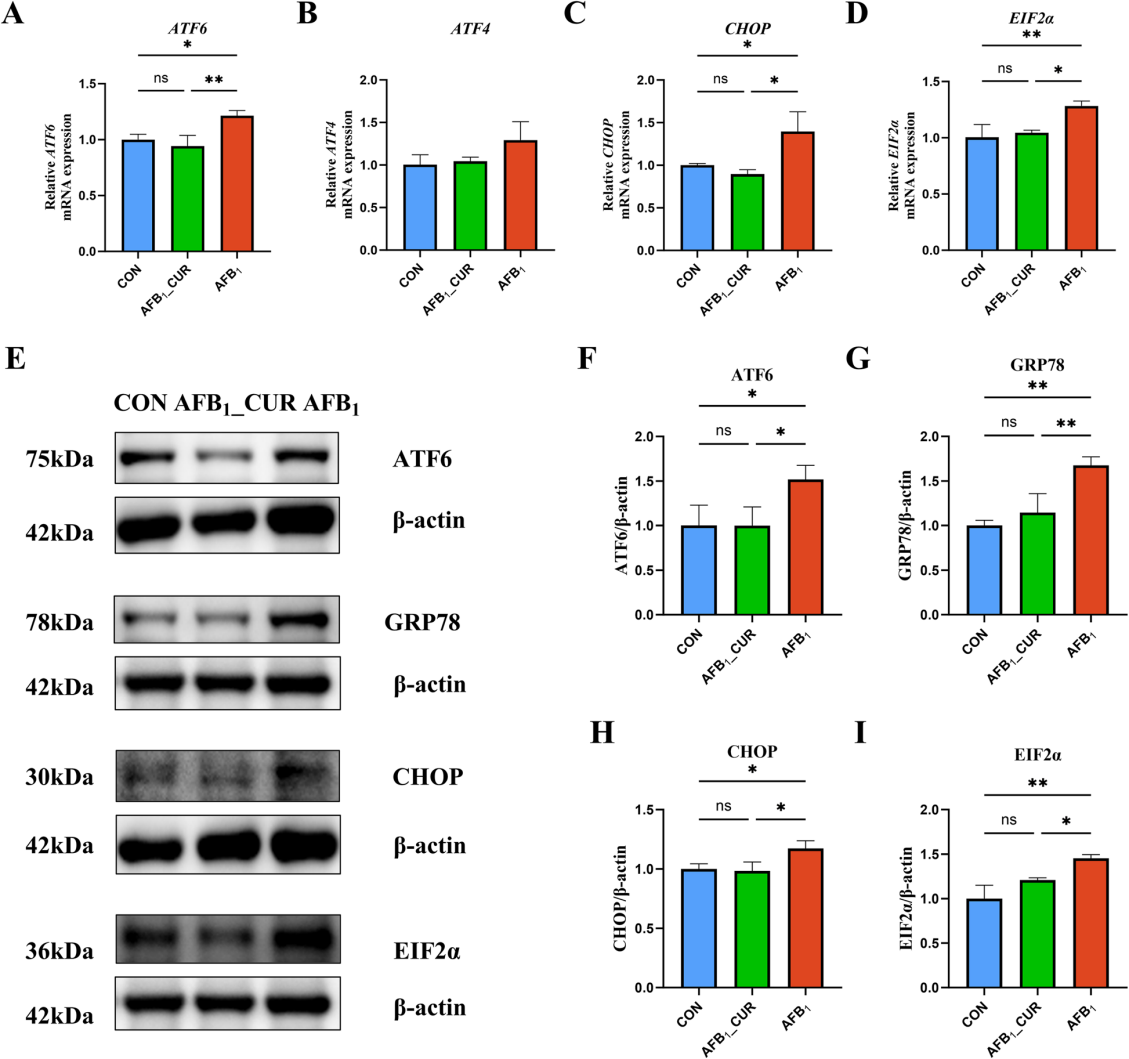
CUR regulates the mRNA (*n* = 6) and protein (*n* = 3) expression of the genes related to the ERS signaling pathway. **A**–**D** Relative mRNA expression of genes related to the ATF6 pathway in renal endoplasmic reticulum stress. *EIF2α*, eukaryotic translation initiation factor 2 alpha ; *CHOP*, DNA damage inducible transcript 3. **E**–**I** Expression of proteins related to ATF6/GRP78 pathway. All data are presented as mean ± SD. ^*^P < 0.05, ^**^P < 0.01; ns, not significant

Oxidative stress and ERS interact, and ERS is induced by AFB_1_ and relieved by CUR. The results confirmed that the relative expression of the ERS genes eukaryotic translation initiation factor 2 alpha (*EIF2α*; *P* < 0.01) and DNA damage-inducible transcript 3 (*CHOP*; *P* < 0.05) markedly increased in the AFB_1_ group and that the expression of the other pathway-related genes *ATF6* (*P* < 0.05) and *ATF4* (*P* < 0.05) was activated. Conversely, the expression of *ATF6* and *ATF4* was inhibited by the addition of CUR, and the expression levels of *CHOP* (*P* < 0.05) and *EIF2α* (*P* < 0.05) were significantly lower (Fig. 12A–D).

Like the mRNA expression levels of related pathway genes, the protein expression levels of ATF6 (*P* < 0.05) and GRP78 (*P* < 0.01) were markedly increased by AFB_1_ treatment, and the expression levels of proteins associated with other pathways downstream of CHOP (*P* < 0.05) and EIF2α (*P* < 0.01) were significantly increased. CUR inhibited the expression of the above genes, thereby alleviating the exacerbation of ERS (*P* < 0.01 or *P* < 0.05; Fig. 12E–I).

### NF-κB signaling in the kidney was inhibited by CUR

**Fig. 13:**
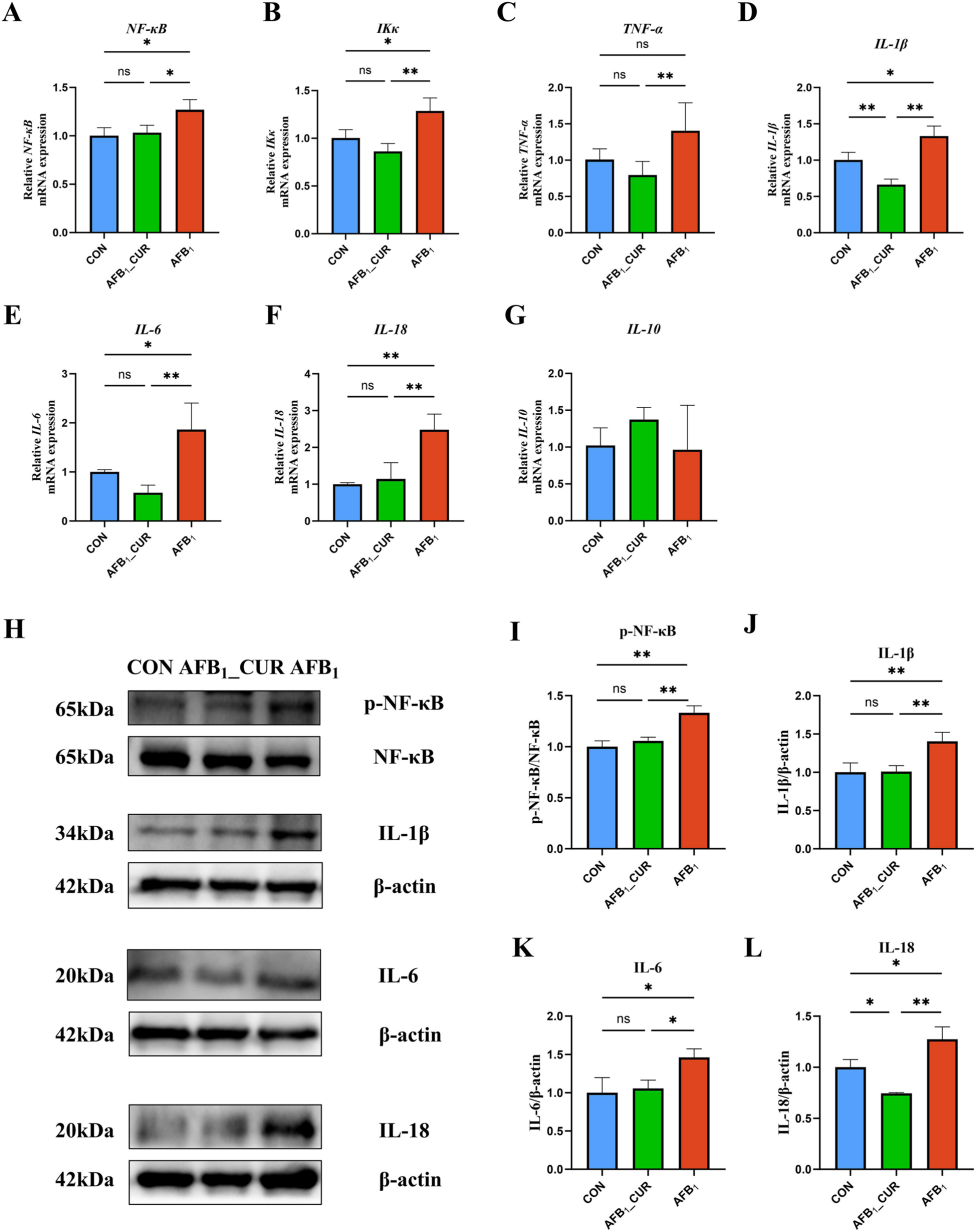
CUR regulates the mRNA (*n* = 6) and protein (*n* = 3) expression of genes related to the jejunal NF-κB signaling pathway. **A**–**G** Relative mRNA expression of NF-κB pathway-related genes in the kidney **H**–**L** The expression of proteins related to the NF-κB pathway in the kidney. All data are presented as mean ± SD. ^*^P < 0.05, ^**^P < 0.01; ns, not significant

Compared with those in the CON group, the relative mRNA expression levels of *NF-κB*, *IKκ*, *IL-1β*, *IL-18* and *IL-6* (*P* < 0.05) in the AFB_1_ group significantly increased but markedly decreased after CUR treatment (*P* < 0.01 or *P* < 0.05; Fig. 13A–G). In brief, AFB_1_ has a harmful effect on renal function, but CUR can relieve this situation.

In this study, the NF-κB signaling pathway was activated by phosphorylation. The protein expression levels of the proinflammatory factors IL-1β, IL-6 and IL-18 (*P* < 0.01) were increased in the AFB_1_ group. The expression levels of p-NF-κB and inflammatory factors were significantly reduced by CUR (*P* < 0.01 or *P* < 0.05; Fig. 13H–L).

### Correlation analysis between differential flora and serum indicators

**Fig. 14:**
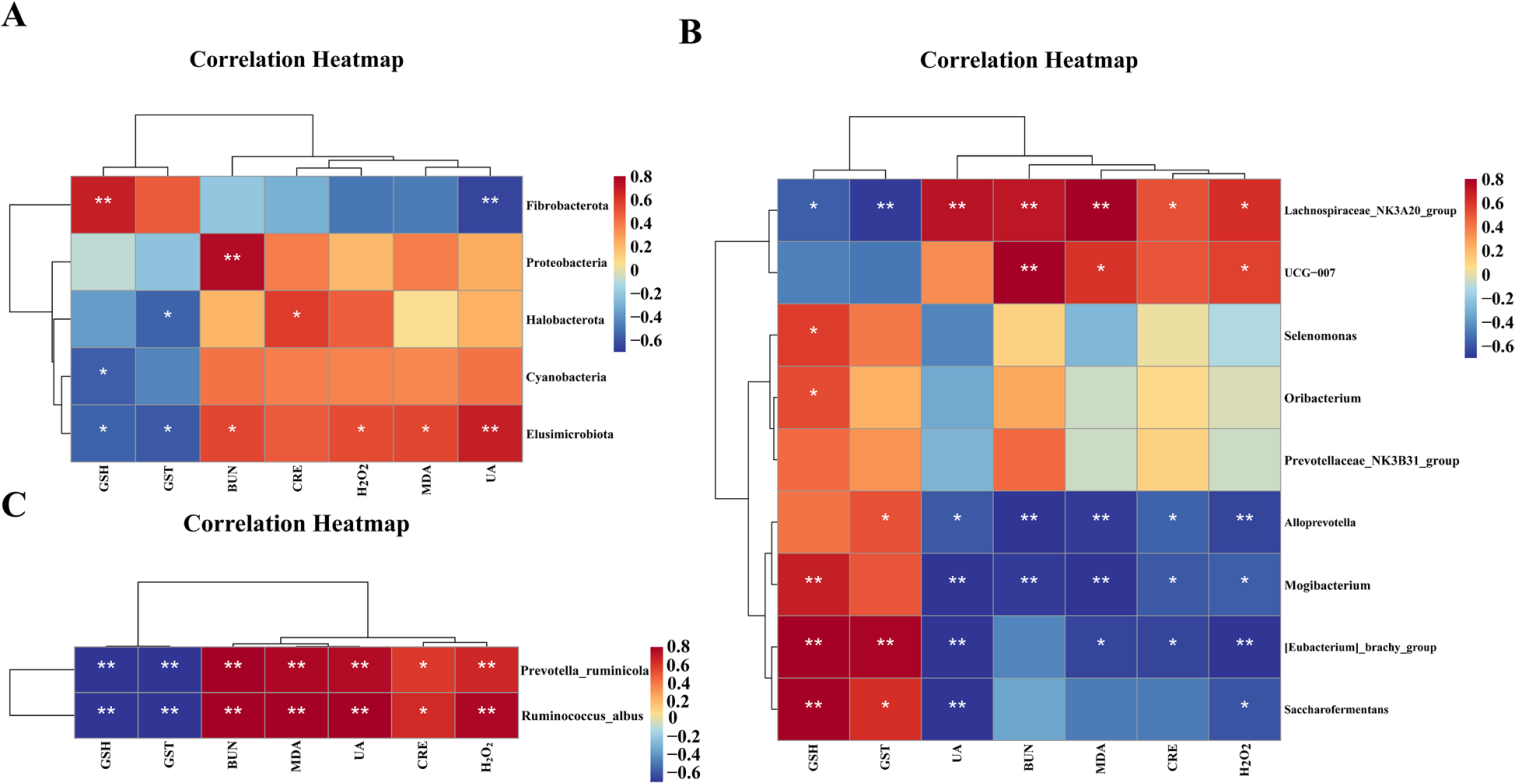
Correlation analysis between differential flora and serum indicators (*n* = 5). **A** Correlation coefficients between phylum-level differences in microorganisms and serum indicators. **B** Correlation coefficients between genus-level differences in microorganisms and serum indicators. **C** Correlation coefficients between species-level differences in microorganisms and serum indicators. ^*^P < 0.05, ^**^P < 0.01

According to the correlation coefficient heatmap results, at the phylum level, the abundance of Fibrobacterota was significantly positively correlated with the GSH content (*P* < 0.01) but significantly negatively correlated with the UA content (*P* < 0.01); moreover, the abundance of Proteobacteria was significantly positively correlated with the BUN content (*P* < 0.01), the abundance of Halobacterota was significantly positively correlated with CRE content (*P* < 0.05), and the abundance of Elusimicrobiota was significantly positively correlated with the BUN content and H_2_O_2_ content (*P* < 0.05; Fig. 14A).

At the genus level, *Lachnospiraceae*_*NK3A20*_*group* was positively correlated with the UA, BUN, CRE and H_2_O_2_ contents (*P* < 0.01 or *P* < 0.05), and *UCG-007* was strongly correlated with the BUN, MDA and H_2_O_2_ contents (*P* < 0.01 or *P* < 0.05). In addition, *Alloprevotella* and [*Eubacterium*]_*ruminantium*_*group* were significantly negatively correlated with H_2_O_2_ (*P* < 0.01) and *Mogibacterium* and *Saccharofermentans* were significantly negatively correlated with H_2_O_2_ content (*P* < 0.05; Fig. 14B).

At the species level, the abundance of *Prevotella* *ruminicola* was strongly correlated with the renal function index (BUN, CRE, and UA; *P* < 0.05) and oxidative stress level (H_2_O_2_ and MDA; *P* < 0.01) and was negatively correlated with reduced GSH content and GST activity (*P* < 0.01), and the abundance of *Ruminococcus* *albus* had the same trend as that of *Prevotella* *ruminicola* (*P* < 0.01; Fig. 14C).

## Discussion

**Fig. 15:**
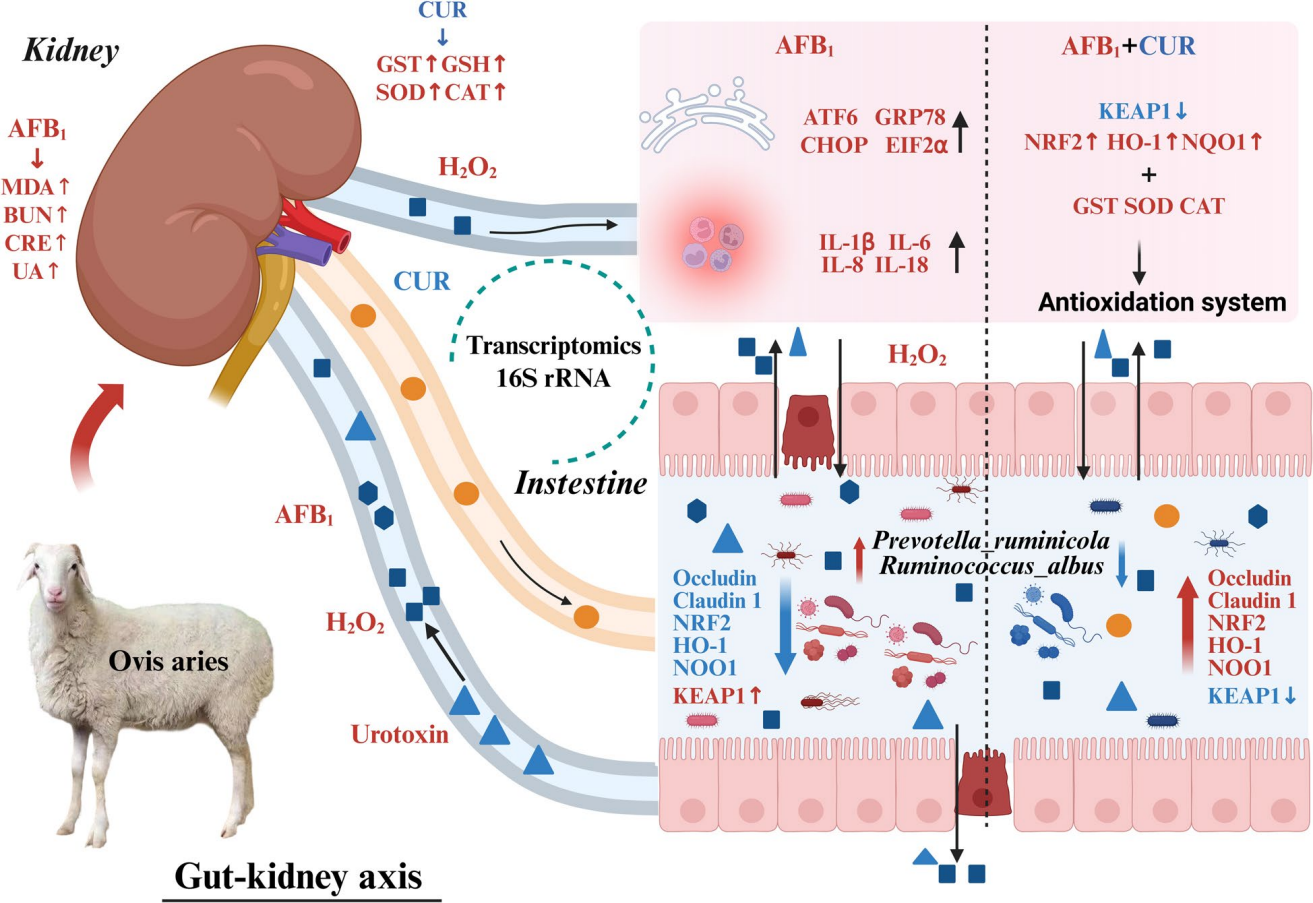
The mechanism of CUR alleviates AFB_1_-exposed sheep’s intestine and kidney injuries via gut-kidney axis. Toxic substances were the key regulating factor in gut-kidney axis. The *Prevotella* *ruminicola* and *Ruminococcus* *albus abundance* were increased by AFB_1_, excessive toxic substances were accumulated and got into intestine and kidney via gut-kidney axis. The ATF6/GRP78 and IL-1β/NF-κB signaling pathways were activated by the double effect of AFB_1_ and H_2_O_2_. However, the NRF2/KEAP1 pathway and antioxidation system were upregulated by CUR which reversing the above trend, gut bacteria were reshaped and toxic substances were decreased in sheep’s gut-kidney axis

The aim of the present study was to elucidate the underlying mechanism through which CUR alleviates AFB_1_-induced damage to the gut-kidney axis in sheep. As a natural herbal extract with a high safety profile, CUR has multiple biological activities, including antioxidation [23], anti-inflammatory [28], antiaging [29], and anti-tumor effects [30]. Previous studies on CUR have focused primarily on its applications in poultry [4, 23, 25] and rodents [20, 31, 32]. In contrast, research on the application of CUR in ruminants remains limited, with few investigations into its regulatory effects on the health of the gut-kidney axis [29]. The present study addressed this knowledge gap and demonstrated that CUR alleviates AFB_1_-induced damage to the gut-kidney axis in sheep through multiple pathways: it inhibits the activity of the ATF6/GRP78 signaling pathway and the NF-κB signaling pathway, upregulates NRF2/KEAP1 signaling pathway, activates the antioxidant system, and reduces AFB_1_-induced injury to the intestines and kidneys of sheep. Consequently, CUR optimizes the structure and function of the intestinal microbiota and safeguards the normal function of the systemic immune system (Fig. 15).

This study verified the positive regulatory effect of CUR on the gut-kidney axis by restoring intestinal microbiota balance, reducing the relative abundance of harmful bacteria and the level of toxic metabolites, enhancing intestinal barrier function, reducing the accumulation of H_2_O_2_ in the intestine and kidney, and promoting the renal metabolic capacity to eliminate waste [33]. The content of H_2_O_2_ decreased as a result of increased activity of antioxidant enzymes, activation of the NRF2 signaling pathway, and the activity of the NF-κB pathway was inhibited by CUR [28, 34]. Activation of the NRF2 signaling pathway and inhibition of the NF-κB signaling pathway align with the results obtained by Jin et al. [25]. Similarly, some harmful anaerobic bacteria lack catalase, resulting in the excessive production of H_2_O_2_. The intestinal integrity is protected by reducing the relative abundance of harmful bacteria and degrading H_2_O_2_ [35, 36]. Because of the presence of H_2_O_2_, the specific mechanism through which CUR mitigates kidney injury involves the NF-κB signaling pathway, calcium signaling pathway, and protein processing in the endoplasmic reticulum. Therefore, the goal of this study was to investigate H_2_O_2_ reduction by CUR, which alleviates oxidative stress, ERS and inflammation, thus reducing apoptosis in the intestine and kidney of sheep via the gut-kidney axis.

The relative height of intestinal villi can reflect the growth of animals, the depth of crypts can reflect the activity and function of stem cells, and intestinal stem cells can respond to stress damage. AFB_1_ reduced the relative villus height and crypt depth of the intestine in sheep, whereas CUR alleviated the effects of AFB_1_ and increased occludin and claudin 1 expression to maintain the balance of gut microorganisms in sheep, thereby improving the function of the intestinal barrier. These findings are consistent with the conclusions of Zhang et al. [37], who reported that CUR alleviates AFB_1_-induced intestinal injury in broilers. The kidney is the second target organ through which AFB_1_ affects the excretion of toxic substances from the body. CUR nanoparticles significantly reduced the serum levels of CRE, UA and BUN and improved the pathological damage to tubules and glomeruli, which affected the filtration function of the glomerulus. These findings are consistent with those of previous studies in which the contents of CRE and BUN were decreased by CUR [38]. HE is recognized as an important phenotypic feature for evaluating injury extent in sheep. Compared with those in the CON group, the villus height and crypt depth in the intestine were lower in the AFB_1_ group but were markedly greater after CUR treatment. H_2_O_2_ can induce oxidative stress, and the effects of oxidative stress on mitochondrial function have been explored [39]. Superoxide anion (O^2−^) scavenging activity is formed by the release of electrons with NADH, which is converted to H_2_O_2_ in the mitochondria, leading to a increase at the level of ROS and resulting in mitochondrial dysfunction [40]. Sheep’s levels of GSH, GST, CAT and SOD were increased by CUR, which could prevent O^2−^ development of H_2_O_2_ and decrease the H_2_O_2_ and MDA contents, thus protecting the body from H_2_O_2_ damage and playing important roles in the biological antioxidant system. The increase in the activity of antioxidant enzymes in this study was consistent with the findings of Wang et al. [11]. The damage to the intestine and kidney of the sheep was mitigated by CUR, which enhanced gut barrier function and antioxidant activity to lower the contents of H_2_O_2_, MDA and renal toxic substances in the sheep’s intestine and kidney.

The intestinal microbiota is important for regulating the intestinal microenvironment, but the intestinal flora of ruminants is more complex and important. Disorders of the gut microbiota can induce a variety of inflammatory conditions, such as inflammatory enteritis, diabetes, arthritis, and neurological diseases [30]. The microbial balance was positively regulated via reduction of the harmful abundance of Proteobacteria, *Prevotella* *ruminicola* and *Ruminococcus* *albus* in response to CUR. The reduction in the abundance of Proteobacteria and *Desulfovibrio* is consistent with the findings of previous studies [41]. However, the abundance of *Prevotella*_*NK3B311*_*groups* significantly increased by AFB_1_ exposed. The reason may be that beneficial bacteria such as *Oribacterium*, *Saccharofermentans*, and *Selenomonas* can produce short-chain fatty acids, providing energy and nutrients to *Prevotella*_*NK3B311*_*groups*, AFB_1_ inhibits the growth of these beneficial bacteria, while harmful bacteria such as *Proteobacteria* proliferate excessively, leading to a loss of synergistic effects, increased competitive pressure, and a loss of advantage in occupying ecological niches, thus inhibiting their growth. Linear discriminant analysis effect size analysis based on the LDA effect enables the screening of potential biomarkers. In our study, Chr*istensenellaceae*_*R7*_*group*, *UCG*‒*005*, and *Methanobrevibacter* were found to be related to metabolism, indicating that CUR is converted into the active substance dihydrocurcumin to reduce the toxicity of AFB_1_ in the gut microbiota [42]. Biomarkers can illustrate the involvement of microorganisms in metabolic processes, whereas functional prediction refers to the ability of CUR to enhance microbial regulatory functional genes and signaling pathways [32]. On the basis of the results of the Tax4Fun function prediction analysis, the knockout genes are enriched mainly in pyruvate-ferredoxin/flavonoid toxin oxidoreductase, antitoxin, ferrous transporter, β-galactosidase, Ca^2+^ transporter ATPase, and glutamine synthase; therefore, CUR alleviates the damage caused by AFB_1_ to the intestine and may play a role through the above genes; therefore, the following verification tests focused mainly on oxidation and reduction reactions and oxidoreductase and NF-κB signaling pathways. Consequently, the functional prediction is the same as the increase in serum antioxidant enzyme activity in the CUR group; thus, CUR possibly alleviate the toxicity of AFB_1_ through the biological antioxidant system and reduce the amount of ROS generated by anaerobic bacteria.

The metabolites of the gut microbiota are closely connected with the host immunometabolism system and regulate physiological metabolic processes through different microorganisms and different chemicals via the host–microbial immunometabolism axis, thus having important effects on animal health and disease occurrence [43]. H_2_O_2_ production is not unique to eukaryotic host cells, and many microorganisms can also produce H_2_O_2_ [44]. *Prevotella* *ruminicola* and *Ruminococcus* *albus* may survive in an anaerobic environment and are positively connected with toxic substances such as H_2_O_2_, BUN, CRE and UA, indicating that CUR decreases H_2_O_2_ content by suppressing the improvement of deleterious anaerobic bacteria. H_2_O_2_ is among the main forms of ROS, and excess H_2_O_2_ can destroy cellular DNA, lipids and proteins, causing severe cell damage and systemic inflammation under stress [45]. Erttmann et al. [33] used a H_2_O_2_-induced colitis model and reported that inflammatory factors (such as TNF-α and IL-6) produced in the colon can enter the circulation and induce inflammation, endothelial dysfunction, and microvascular damage in distant organs such as the kidney. Uric acid crystals can activate inflammation and promote NADPH oxidase to produce H_2_O_2_, thereby inducing oxidative stress and inflammatory responses, resulting in damage to the animal body [46, 47]. In this study, compared with those in the AFB_1_ group, the serum BUN and UA levels in the CUR group were significantly lower, and the abundance of *Elusimicrobiota* in the AFB_1_ group was significantly greater. CUR inhibits the growth of *Elusimicrobiota* by promoting the proliferation of beneficial bacteria, thereby reducing hydrogen production by *Elusimicrobiota*. Consequently, the timely generation of H_2_O_2_ is impaired, which alleviates H_2_O_2_-mediated oxidative stress and the activation of ERS. If excessive UA cannot be excreted through the intestine, UA deposition aggravates kidney function damage; moreover, disturbance of the intestinal microflora causes inflammatory damage to the intestine. However, Fibrobacterota was significantly positively correlated with the GSH content, and CUR improved glutathione metabolism, thereby enhancing the ability of sheep to resist AFB_1_ toxicity. The high abundance of *Oscillospira* is in agreement with the research outcomes of Sun et al. [41, 48]. In conclusion, CUR inhibited oxidative stress in the intestine as well as kidney injury through the gut-kidney axis, thereby alleviating crosstalk damage to the intestine and kidney.

Through the changes in the above indices, the specific mechanism of CUR was further analyzed in combination with the transcriptome. Transcriptome analysis explored CUR targets and enriched pathways to verify the underlying molecular mechanism, providing theoretical support for the application of CUR. Disruption of redox reactions leads to the generation of oxidative stress, with a large accumulation of ROS triggering inflammation [49]. In this study, the genes related to redox reactions, oxidase activity, ERS and inflammation in the CUR group. The correlation analysis of oxidative stress, ERS and inflammation revealed that *NRF2* was significantly positively correlated with *IL-18* and* RELA* and *ATF6* was strongly connected with *EIF2A* and *ATF4*. Similarly, these genes were related to the Ca^2+^, NRF2, ATF6 and NF-κB signaling pathways. Ca^2+^ is generated by oxidative stress through ROS-sensitive Ca^2+^ channels and upregulated ROS to induce inflammation and apoptosis [50]. Similarly, Ca^2+^ worsened ERS at the level of internal flow, and ERS induced oxidative stress inversely, amplifying inflammation, which in turn led to apoptosis [51]. In the study, KEGG analysis revealed enrichment of NF-κB, protein processing in the endoplasmic reticulum and glutathione metabolism. CUR inhibited the activation of NF-κB, improved glutathione metabolism, promoted the normal operation of redox reactions, and reduced the misfolding of proteins in the endoplasmic reticulum, thus exerting a protective effect on the kidney. This observation aligns with the results reported by Abah et al. [52] and Chen et al. [53] showing that CUR can exert targeted regulation of ERS via PTEN and IRE1 signaling pathways. Therefore, the results of the transcriptome analysis provide a theoretical basis for the mechanism through which CUR regulates the gut-kidney axis.

The mechanism underlying intestinal and kidney injuries was confirmed to involve the inhibition of ATF6/GRP78 and IL-1β/NF-κB signaling pathways and the activation of NRF2/KEAP1 signaling pathway by CUR via the gut-kidney axis. Oxidative stress is an important upstream trigger of ERS, which further activates inflammation through NF-κB signaling. In this study, NRF2 is a key regulator for antioxidant enzyme system, and the NRF2 pathway was inhibited by the production of H_2_O_2_. CUR dissociates NRF2 from KEAP1, thereby activating downstream genes *HO-1* and *NQO1*, and subsequently enhancing antioxidant enzyme activity. The biological antioxidant system acts as a protective barrier against oxidative stress and is activated by CUR to upregulate the activity of GST, SOD and CAT to reduce the H_2_O_2_ and MDA contents. GST is part of the detoxification system and catalyzes the conjugation of metabolic products with the thiol group of GSH to form hydrophilic substances, which are then easily excreted from the body [22, 54]. SOD catalyzes the conversion of O^2−^ to H_2_O_2_ and O_2_, while CAT catalyzes the conversion of H_2_O_2_ into water and O_2_; these enzymes are able to reduce H_2_O_2_ content together, thereby protecting cells from oxidative damage [55]. Similarly, the ERS pathway is at the core of the ROS-UPR-inflammatory factor mechanism, indicating that it reversely induces oxidative stress, which further amplifies inflammation and apoptosis, ultimately forming a closed-loop cycle [56, 57]. The UPR signaling pathway perceives and regulates protein folding capacity to restore endoplasmic reticulum homeostasis [53], and ATF6/GRP78 is one of the key components in the UPR pathway. ATF6/GRP78 is activated by ROS [58], which are divided into GRP78, inducing ERS and the relaxation of inflammatory factors (IL-1β, IL-6 and TNF-α) [59] following NF-κB activation and apoptosis. In the study, the expression of CHOP was upregulated by ATF6, which promoted the expression of BAX and caspase 3. CUR alleviated the harmful effects of the gut-kidney axis and activated NRF2 signaling and downstream genes, thereby promoting the protective effect of the antioxidant system and inhibiting the expression of NF-κB, ATF6, GRP78, CHOP and EIF2α in sheep. On the one hand, the integrity of the intestinal barrier improved, and the balance of the gut microbiome was restored by CUR treatment, restoring the redox electron balance in sheep, inhibiting the activity of the ATF6/GRP78 and NF-κB signaling pathways to mitigate to the intestine and kidney injuries, and enhancing the protective effect of the antioxidant system on the gut-kidney axis. On the other hand, the homeostasis of arginine biosynthesis relies on the gut-kidney axis coordination. The gut synthesizes citrulline through the glutamic acid pathway, which is then converted into arginine by the kidney [60] and transported to other tissues via blood circulation, maintaining animal health. Therefore, CUR restores the homeostasis of arginine biosynthesis, providing energy repair for inflammatory damage in the gut-kidney axis.

## Conclusions

CUR can alleviate AFB_1_-induced intestinal and kidney injuries by improving intestinal barrier function, inhibiting the activity of the ATF6/GRP78 and IL-1β/NF-κB signaling pathways, reducing apoptosis and inflammatory damage, and regulating the intestinal microbiota via the gut-kidney axis. The protective effect of CUR on the intestines and kidneys is associated with decreased levels of *Prevotella* *ruminicola*, *Ruminococcus* *albus* and toxic substances, as well as the activation of the NRF2/KEAP1 pathway to regulate the antioxidant system.

## Supplementary Information

Additional file 1: Table S1 Composition and nutritional levels of the basal diet for sheep. Table S2 Primer sequences for gene amplification. Table S3 Primary antibodies information.

Additional file 2. The Western blot images.

## Data Availability

The 16S rRNA sequencing and transcriptomics datasets have been deposited in the NCBI SRA database (PRJNA1332410) and NCBI GEO database (GSE309094), respectively.

## Abbreviations

AFB_1_: Aflatoxin B_1_
CUR: Curcumin
ERS: Endoplasmic reticulum stress
ROS: Reactive oxygen species
UPR: Unfolded protein response
ATF6: Activating transcription factor 6
GRP78: Glucose-regulated protein, 78 kDa
NF-κB: Nuclear factor of kappa light polypeptide gene enhancer in B cells
IL-1β: Interleukin 1 beta
BCL2: BCL2 apoptosis regulator
PI3K: Phosphatidyqinositol‐3 kinase
MAPK: Mitogen-activated protein kinase
Erk: Extracellular regulated protein kinases
TLR4: Toll like receptor 4
DM: Dry matter
TMR: Total mixed ration
GSH: Glutathione
GST: Glutathione S-transferase activity
CAT: Catalase
SOD: Superoxide dismutase
BUN: Urea nitrogen
CRE: Creatinine
UA: Uric acid
VCR: Villi crypt rate
KEGG: Kyoto Encyclopedia of Genes and Genomes
ZO-1: Tight junction protein 1
Cytc: Cytochrome C
BAX: BCL2 associated X
NRF2: Nuclear factor erythroid 2-related factor 2
KEAP1: Kelch like ECH associated protein 1
HO-1: Heme oxygenase 1
NQO1: NAD(P)H quinone dehydrogenase 1
SOD1: Superoxide dismutase 1
SOD2: Superoxide dismutase 2
IL-18: Interleukin 18
IL-6: Interleukin 6
IKκ: Inhibitor of nuclear factor kappa B kinase
TNF-α: Tumor necrosis factor α
GSTA1: Glutathione S-transferase alpha 1
PRDX1: Peroxiredoxin 1
EIF2AK2: Eukaryotic translation initiation factor 2 alpha kinase 2
ATF4: Activating transcription factor 4
RELA: RELA proto-oncogene
CXCL9: C-X-C motif chemokine ligand 9
IL6R: Interleukin 6 receptor
CASP9: Caspase 9
EIF2α: Eukaryotic translation initiation factor 2 alpha
CHOP: DNA damage inducible transcript 3
O^2-^: Superoxide anion
LDA: Linear discriminant analysis
