# Supplementary material for: Curcumin mitigated aflatoxin B1-induced endoplasmic reticulum stress and gut-kidney axis damage in sheep by regulating the ATF6/GRP78 and IL-1β/NF-κB signaling pathways
Source: J Anim Sci Biotechnol. 2026 Apr 21;17:74. doi: 10.1186/s40104-026-01382-2 (PMC13097883; doi:10.1186/s40104-026-01382-2)
Supplement: Supplementary file 1 — Additional file 1: Table S1 Composition and nutritional levels of the basal diet for sheep. Table S2 Primer sequences for gene amplification. Table S3 Primary antibodies information. [file 40104_2026_1382_MOESM1_ESM.docx]

**Table S1** Composition and nutritional levels of the basal diet for sheep (DM basis, %)

| **Ingredients** | **Content%** | **Nutrient levels** | **Content** |
| --- | --- | --- | --- |
| Corn | 22.93 | DM^2^ (%) | 89.65 |
| Soybean meal | 18.50 | ME (MJ/kg) | 8.49 |
| Cottonseed meal | 5.00 | CP (%) | 17.40 |
| Wheat bran | 3.00 | DP (%) | 2.85 |
| Corn DDGS | 6.32 | Ca (%) | 0.57 |
| Corn germ meal | 5.00 | TP (%) | 0.39 |
| Soybean oil | 0.70 | EE (%) | 3.47 |
| Limestone | 0.90 | Ash (%) | 5.90 |
| Premix^1^ | 1.00 | NDF (%) | 29.93 |
| NaCl | 1.00 | CF (%) | 12.87 |
| Soybean hulls | 3.00 | ADF (%) | 15.00 |
| Beet molasses | 2.10 | NFE (%) | 44.88 |
| NaHCO₃ | 0.35 |  |  |
| Na₂SO₄ | 0.20 |  |  |
| Chinese leymus | 30.00 |  |  |
|  | 100.00 |  |  |

^1^Each kg of the premix diet provided the following component: Cu: 12 mg; Fe: 90 mg; Mn: 45 mg; Zn: 43 mg; Se: 0.2 mg; I: 0.7 mg; Co: 0.2 mg; VA: 8000 IU; VD: 2000 IU; VE: 80 IU.

^2^The value of NDF, ADF and CP are actual measurement, other contents are calculated value.

This nutritional information table refers to the experimental designs of other members of the research team.

**Table S2** Primer sequences for gene amplification

| **Gene name^a^** | | **NCBI ID** | **Sequence of primer (5'→3')** | **Product size, bp** | | | **Tm^b^,℃** |  |
| --- | --- | --- | --- | --- | --- | --- | --- | --- |
| *β-actin* | NM_001009784.3 | | F: AGATCAAGATCATCGCGCCC  R: ACTCCTGCTTGCTGATCCAC | | 108 | 54.7  53.7 | |  |
| *ZO-1* | XM_042235170.2 | | F: CAAGAGGAGGCTGTGGGTAA  R: TCTTGCTCCTGTGCAGTCTC | | 116 | 58.7  59.1 | |  |
| *occludin* | XM_015101255.4 | | F: AGGACGACTGGATCAGGGAA  R: CTGCAGGCCAGTGTCAAAAC | | 86 | 58.0  58.4 | |  |
| *claudin 1* | NM_001185016.1 | | F: GACGACGAGGCACAGAAGAT  R: TACCATGCTGTGGCAACCAA | | 92 | 57.6  58.6 | |  |
| *Cytc* | XM_004014896.5 | | F: TGCTGCCAAGAATGTCGTCT  R: TTGCCTCGCCATTTGAAAGC | | 135 | 59.2  60.1 | |  |
| *caspase 3* | XM_060406953.1 | | F: AGCAAATCAGTGGACTCCGG  R: GCTCGTGAAGGTTTCCCTGA | | 171 | 58.2  58.4 | |  |
| *caspase 9* | XM_060396596.1 | | F: TCCTGGAGGGATCCCAAGAG  R: GAAGGTCCTCACAGTGAGCC | | 85 | 58.8  58.9 | |  |
| *BAX* | XM_027978592.3 | | F: CATGGGCTGGACATTGGACT  R: AAAGTAGGAGAGGAGGCCGT | | 88 | 60.3  59.8 | |  |
| *BCL2* | XM_012103831.5 | | F: CTGGATCCAGGACAACGGAG  R: CAGACTGAGCAGTGCCTTCA | | 109 | 58.9  59.3 | |  |
| *NRF2* | XM_015093345.4 | | F: TGATGGACTTGGAGCTGCCC  R: AGCTCATGCTCCTTCTGTCG | | 142 | 60.6  57.4 | |  |
| *KEAP1* | XM_027969637.3 | | F: AGAGAAACGAGTGGCGGATG  R: CCCCCGCAGCATAGATACAG | | 93 | 57.6  58.3 | |  |
| *HO-1* | XM_027967703.3 | | F: GCTACCTGGGCGACCTGTC  R: GCTGGCGATATTGGGGAAAGTG | | 116 | 61.8  59.1 | |  |
| *NQO1* | XM_004015102.6 | | F: AGGATGGAAGAAACGCCTGG  R: GCTCATCCTGCACCTCCTTT | | 122 | 56.1  55.9 | |  |
| *SOD1* | NM_001145185.2 | | F: GGTGATCATGGGTTCCACGT  R: TTCACATTGCCCAGGTCTCC | | 143 | 60.2  61.1 | |  |
| *SOD2* | NM_001280703.1 | | F: GGCCTACGTGAACAACCTCA  R: ACTTTAACGCAGGCTGCAGA | | 101 | 58.5  59.6 | |  |
| *NF-κB* | XM_012179609.5 | | F: TTTCAACCGGAGATGCCACT  R: GGCCTTCACACACATAACGG | | 100 | 60.5  60.9 | |  |
| *IKκ* | XM_042241396.2 | | F: TGGTCCTTATGAGGCCGGTA  R: GAGTCCCTGAGAGATGGGGT | | 143 | 58.6  60.1 | |  |
| *TNF-α* | NM_001024860.1 | | F: ACGAACCCATCTACCAGGGA  R: AGACTCGGCATAGTCCAGGT | | 98 | 61.2  58.2 | |  |
| *IL-1β* | NM_001009465.2 | | F: ACAGATGAAGAGCTGCACCC  R: AGACATGTTCGTAGGCACGG | | 161 | 56.9  57.7 | |  |
| *IL-6* | NM_001009392.1 | | F: TTCACAAGCGCCTTCAGTCC  R: GGGGTAGGGAAAGCAGAAGTC | | 74 | 61.3  62.1 | |  |
| *IL-18* | XM_012095263.4 | | F: AAATGGCGACCTGGAATCAGA  R: ACCTCTAGTGAGGCTGTCCTT | | 199 | 56.4  57.5 | |  |
| *IL-10* | NM_001009327.1 | | F: GTCATCGTTTTCTGCCCTGC  R: CCCCTCTCTTGGAGCATATTGA | | 82 | 59.8  60.2 | |  |
| *ATF6* | XM_042256929.1 | | F: TAAAGGTCAGACCGTGGTGC  R: GCAACAGCAAGGACTGGTTG | | 93 | 56.5  58.1 | |  |
| *ATF4* | | NM_001142518.1 | | F: AGGAGGATGCCCACTCAGAT  R: TCTCCAGGAGGGTCGTAAGG | | 172 | 58.3  59.6 | |
| *CHOP* | | XM_060412903.1 | | F: AGTGGCACAGCTAGCTGAAG  R: GGTCAATTAGAGCTCGGCGA | | 95 | 57.1  57.1 | |
| *EIF2α* | | XM_027959332.3 | | F: CAGACGCTGCACTACATTGC  R: CACAGGATCGCACTTCAGGT | | 168 | 56.3  56.8 | |

^a^*β-actin*, actin beta; *ZO-1*, tight junction protein 1; *Cytc*, cytochrome C; *BAX*, BCL2 Associated X; *BCL2*, BCL2 apoptosis regulator; *NRF2*, nuclear factor erythroid 2-related factor 2; *KEAP1*, kelch like ECH associated protein 1; *HO-1*, heme oxygenase 1; *NQO1*, NAD(P)H quinone dehydrogenase 1; *SOD1*, superoxide dismutase 1; *SOD2*, superoxide dismutase 2; *NF-κB*, nuclear factor of kappa light polypeptide gene enhancer in B cells; *Ikκ*, inhibitor of nuclear factor kappa B kinase subunit beta; *TNF-α*, tumor Necrosis Factor α; *IL-1β*, interleukin 1 beta; *IL-6*, interleukin 6; *IL-18*, interleukin 18; *IL-10*, interleukin 10; *ATF6*, activating transcription factor 6; *ATF4*, activating transcription factor 4; *CHOP*, DNA damage inducible transcript 3; *EIF2α*, eukaryotic translation initiation factor 2 alpha kinase 3.

^b^Tm, melting temperature.

**Table S3** Primary antibodies information

| **Antibody name** | **Factory** | **Item number** | **Dilution ratio** |  |
| --- | --- | --- | --- | --- |
| β-actin | Proteintech | 81115-1-RR | 1:25000 |  |
| occludin | Proteintech | 13409-1-AP | 1:1000 |  |
| claudin 1 | Proteintech | 13050-1-AP | 1:5000 |  |
| BAX | Proteintech | 50599-2-Ig | 1:5000 |  |
| BCL2 | Bioss | bsm-61074R | 1:2000 |  |
| caspase 3 | Proteintech | 19677-1-AP | 1:1000 |  |
| NRF2 | Proteintech | 16396-1-AP | 1:5000 |  |
| KEAP1 | CST | #8047 | 1:1000 |  |
| HO-1 | Proteintech | 10701-1-AP | 1:5000 | |
| NQO1 | Proteintech | 11451-1-AP | 1:5000 | |
| NF-κB | Bioworld | BS9879M | 1:1000 | |
| p-NF-κB | Affinity | AF2006 | 1:1000 | |
| IL-18 | Proteintech | 27095-1-AP | 1:10000 | |
| IL-1β | Proteintech | 16806-1-AP | 1:5000 | |
| IL-6 | Proteintech | 21865-1-AP | 1:1000 | |
| ATF6 | Proteintech | 24169-1-AP | 1:5000 | |
| GRP78 | Proteintech | 11587-1-AP | 1:6000 | |
| CHOP | Proteintech | bs-1219R | 1:2000 | |
| EIF2α | Proteintech | bs-3613R | 1:1000 | |
