# Supplementary figures and images for: Curcumin mitigated aflatoxin B1-induced endoplasmic reticulum stress and gut-kidney axis damage in sheep by regulating the ATF6/GRP78 and IL-1β/NF-κB signaling pathways
Source: J Anim Sci Biotechnol. 2026 Apr 21;17:74. doi: 10.1186/s40104-026-01382-2 (PMC13097883; doi:10.1186/s40104-026-01382-2)

**The Western Blots images of intestine**
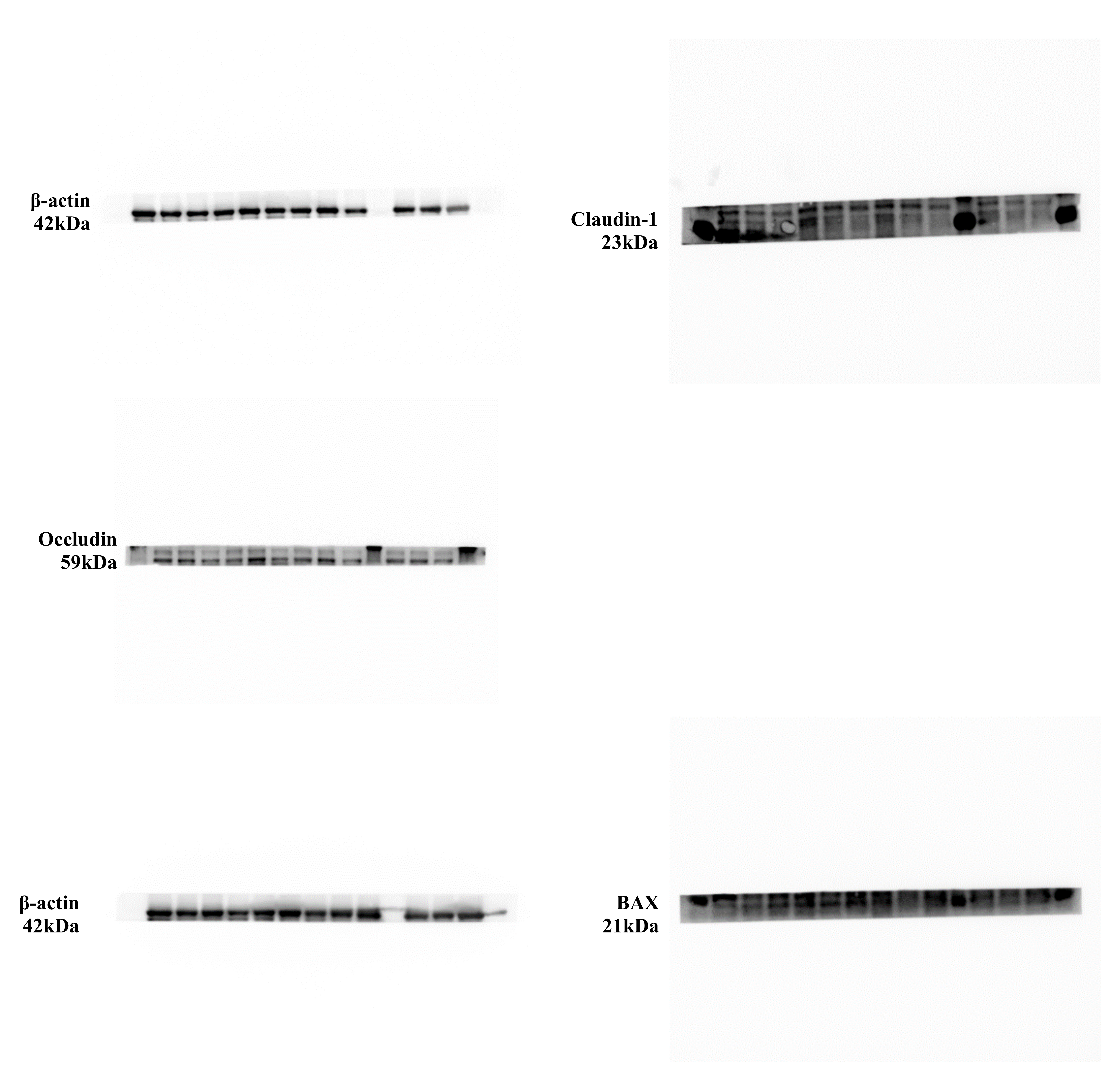


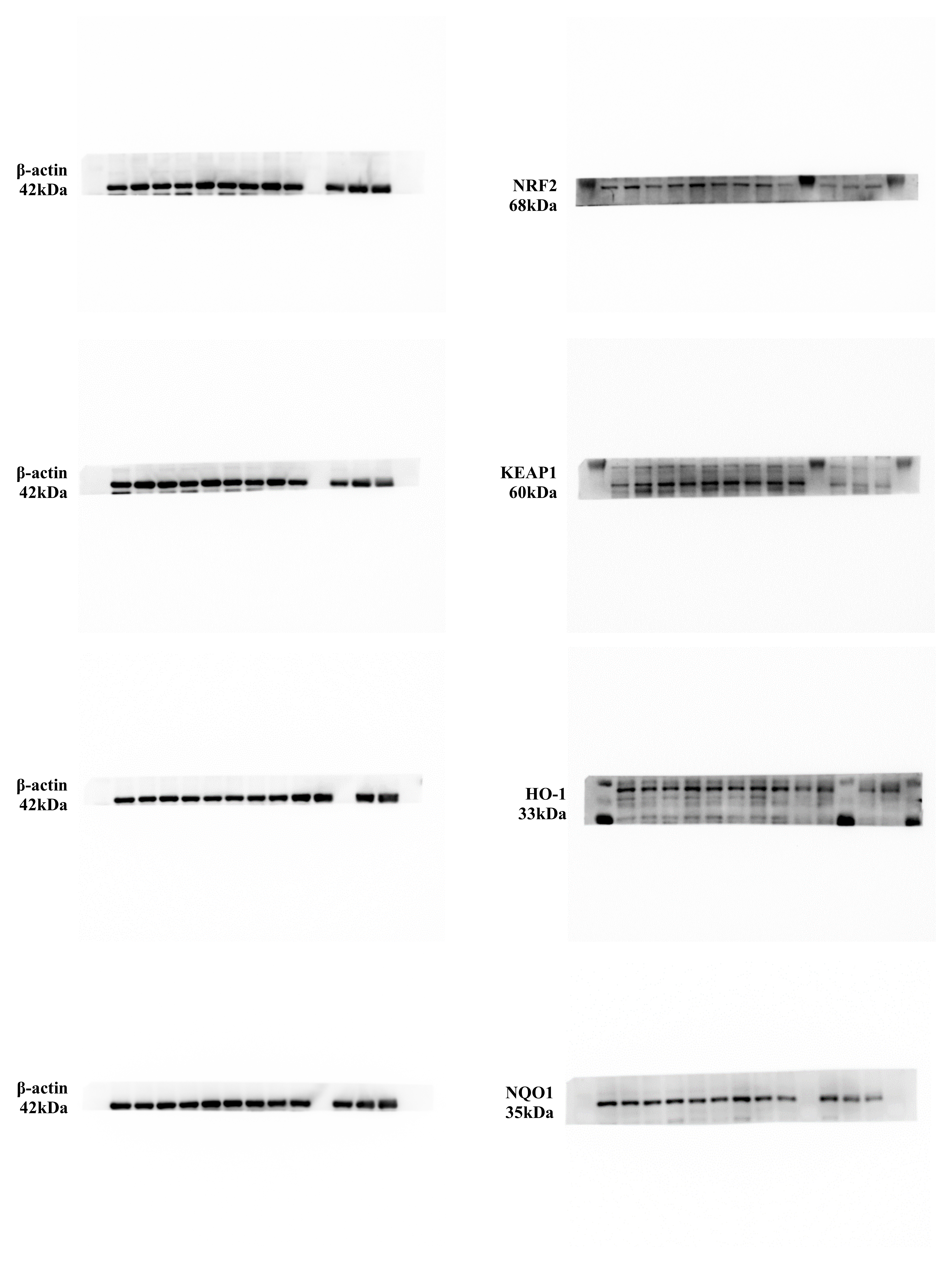


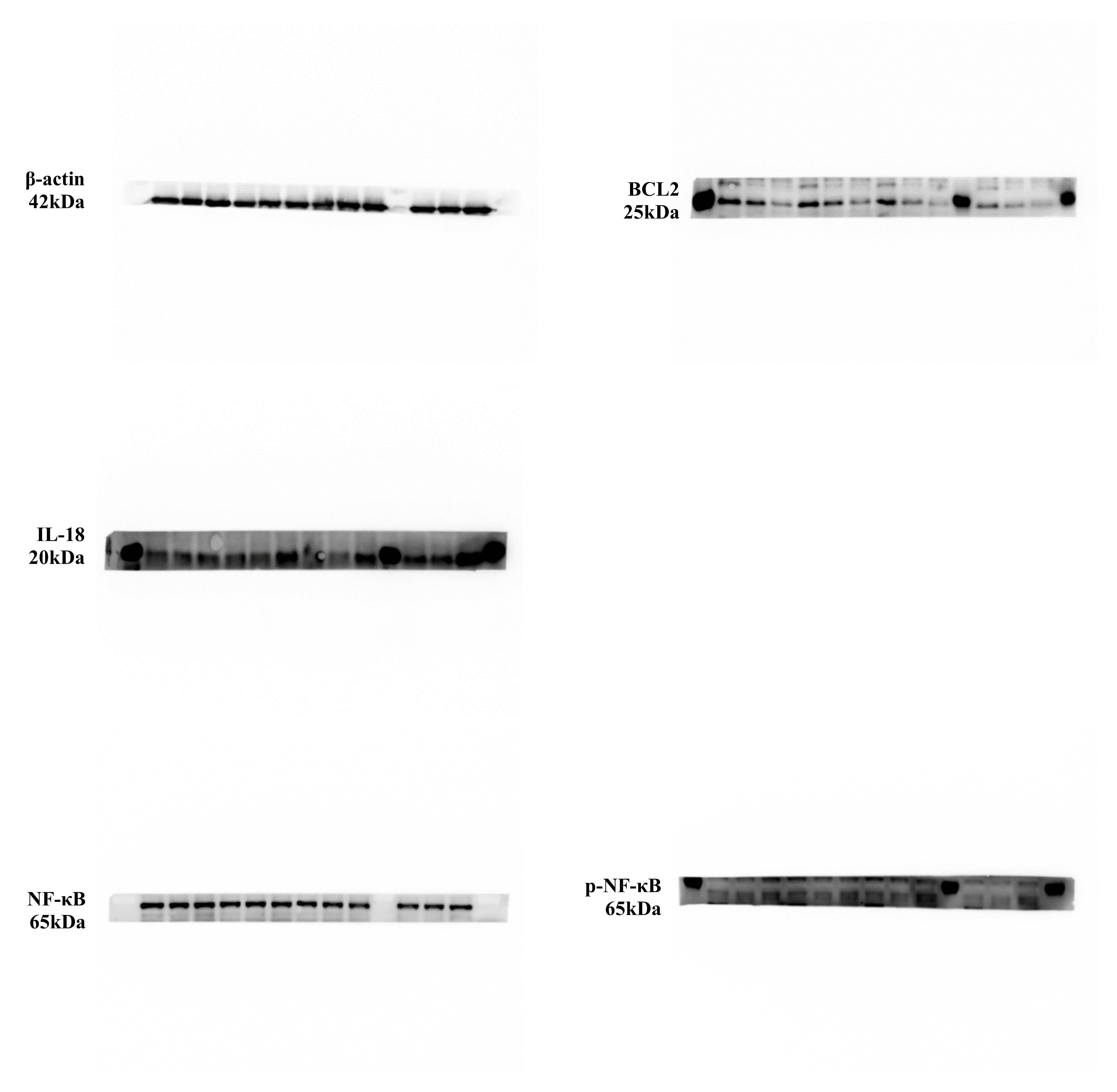


**The Western Blots images of kidney**


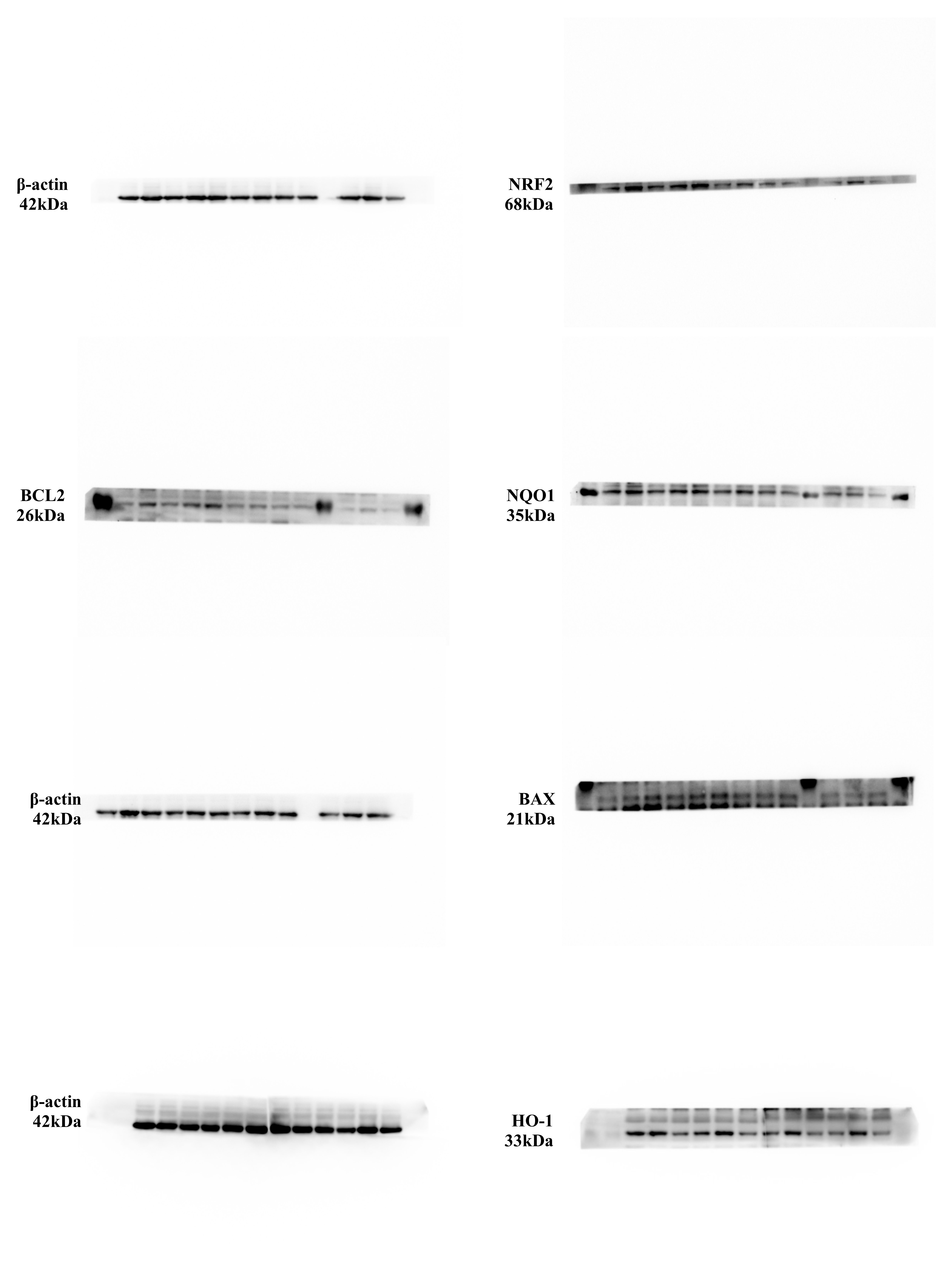


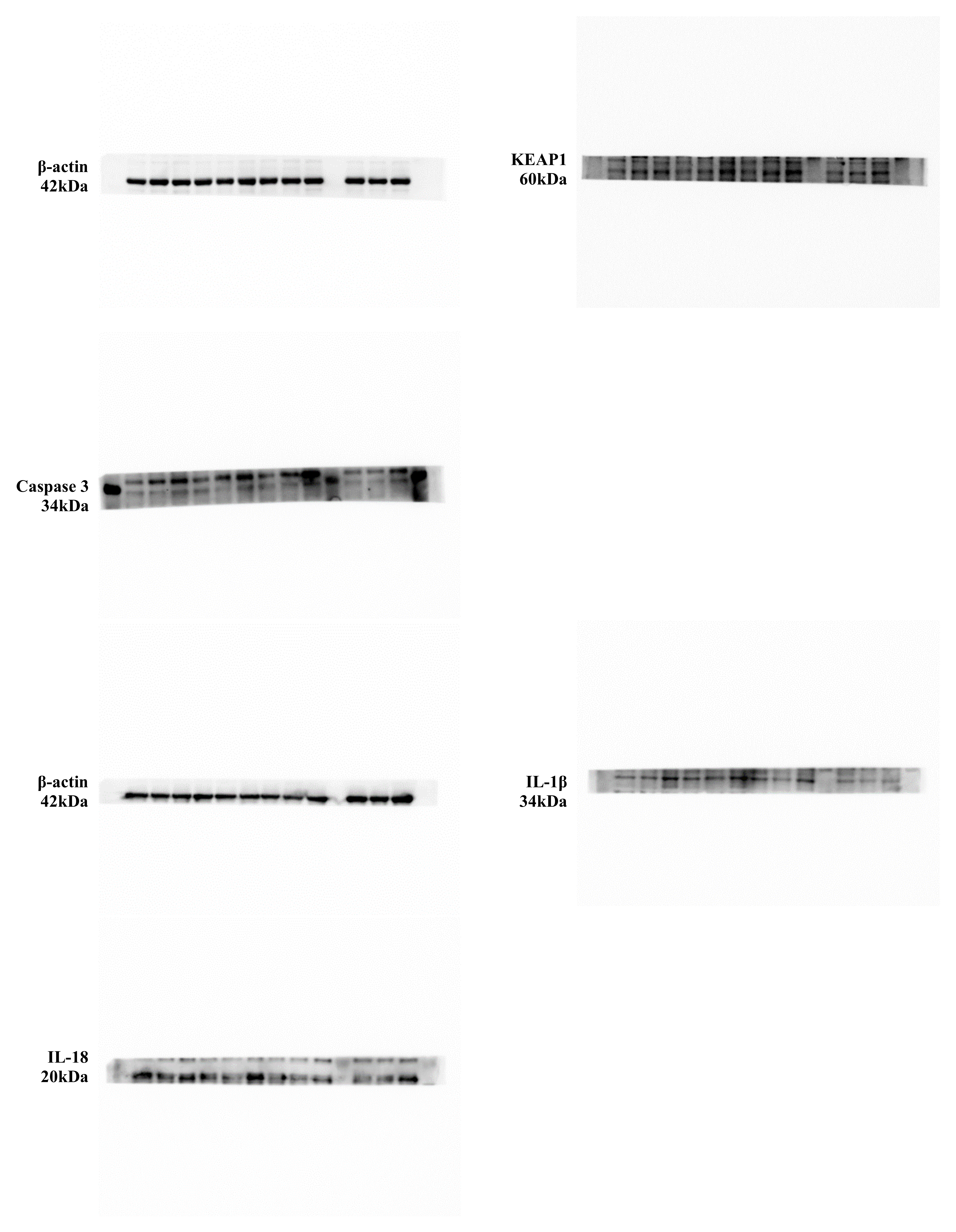


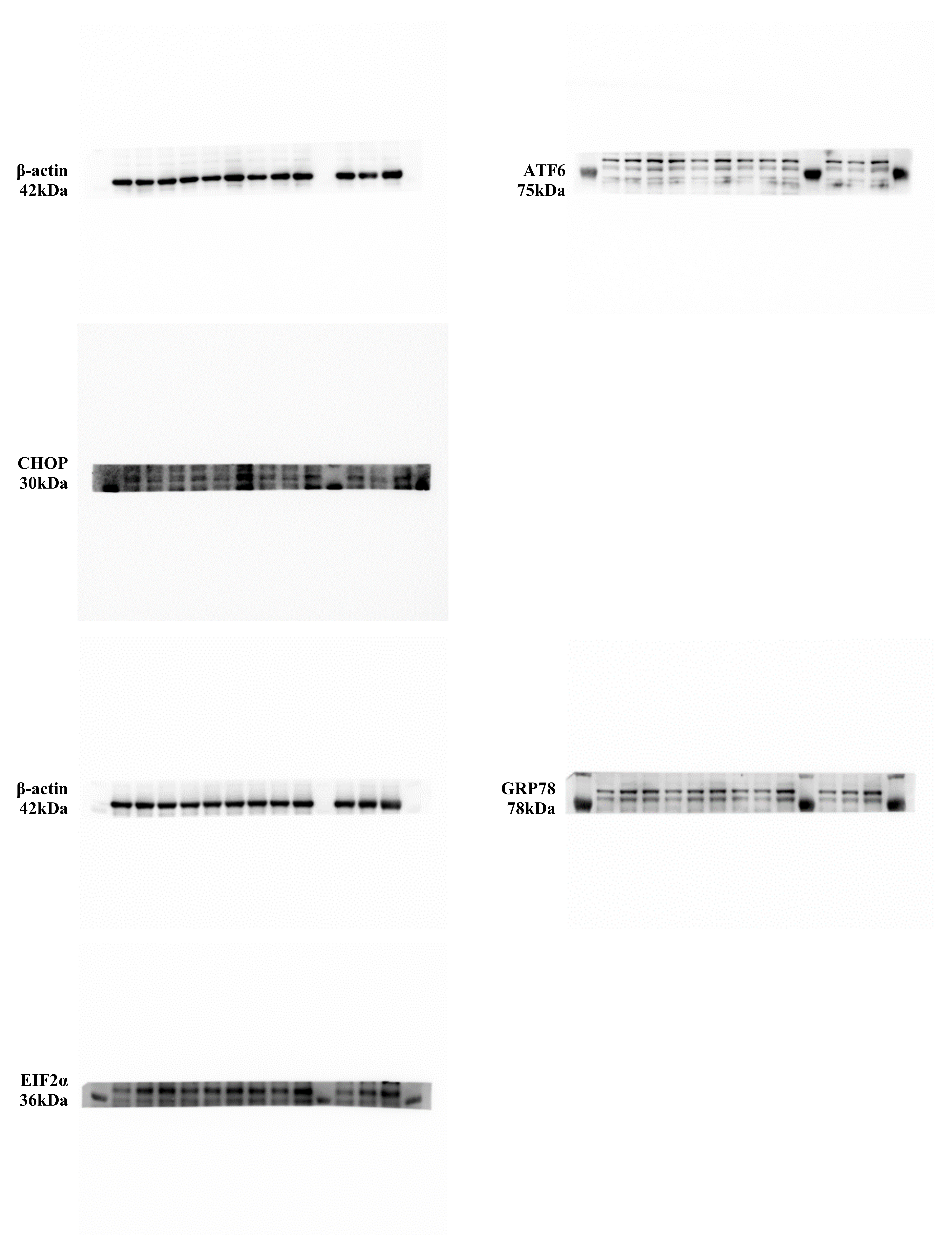


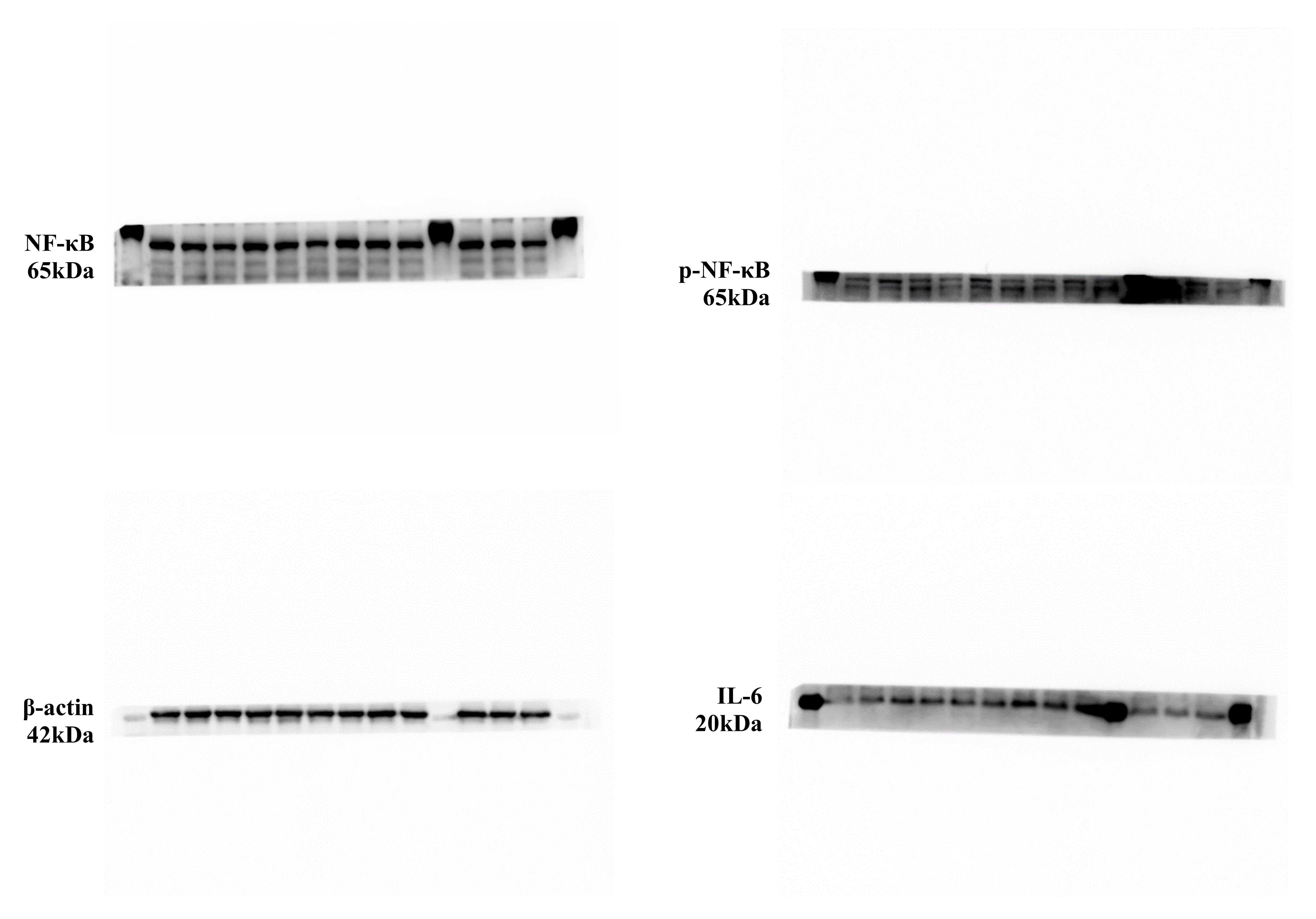

Supplement: Supplementary file 2 — Additional file 2. The Western blot images. [file 40104_2026_1382_MOESM2_ESM.docx]
